# Abscisic Acid Regulates Root Elongation Through the Activities of Auxin and Ethylene in *Arabidopsis thaliana*

**DOI:** 10.1534/g3.114.011080

**Published:** 2014-05-15

**Authors:** Julie M. Thole, Erin R. Beisner, James Liu, Savina V. Venkova, Lucia C. Strader

**Affiliations:** * Department of Biology, Washington University in St. Louis, St. Louis, Missouri 63130; † Department of Biochemistry and Cell Biology, Rice University, Houston, Texas 77005

**Keywords:** mutant screen, plant hormones, epistasis, whole genome sequencing

## Abstract

Abscisic acid (ABA) regulates many aspects of plant growth and development, including inhibition of root elongation and seed germination. We performed an ABA resistance screen to identify factors required for ABA response in root elongation inhibition. We identified two classes of *Arabidopsis thaliana* AR mutants that displayed ABA-resistant root elongation: those that displayed resistance to ABA in both root elongation and seed germination and those that displayed resistance to ABA in root elongation but not in seed germination. We used PCR-based genotyping to identify a mutation in *ABA INSENSITIVE2* (*ABI2*), positional information to identify mutations in *AUXIN RESISTANT1* (*AUX1*) and *ETHYLENE INSENSITIVE2* (*EIN2*), and whole genome sequencing to identify mutations in *AUX1*, *AUXIN RESISTANT4* (*AXR4*), and *ETHYLENE INSENSITIVE ROOT1*/*PIN-FORMED2* (*EIR1/PIN2*). Identification of auxin and ethylene response mutants among our isolates suggested that auxin and ethylene responsiveness were required for ABA inhibition of root elongation. To further our understanding of auxin/ethylene/ABA crosstalk, we examined ABA responsiveness of double mutants of *ethylene overproducer1* (*eto1*) or *ein2* combined with auxin-resistant mutants and found that auxin and ethylene likely operate in a linear pathway to affect ABA-responsive inhibition of root elongation, whereas these two hormones likely act independently to affect ABA-responsive inhibition of seed germination.

Plant growth and development are dynamic processes that allow for adaptation to the environment to which they are restricted. Plants adjust growth in response to internal and external stimuli through the activities of plant hormones. The phytohormones abscisic acid (ABA), auxin, and ethylene play key roles in regulating these growth responses (Vanstraelen and Benková 2012).

ABA controls many growth and stress responses, including seed dormancy onset and maintenance, germination inhibition, stomatal opening, and shoot and root growth (Cutler *et al.* 2010). Both genetic and chemical genomic screens in *Arabidopsis thaliana* have revealed many genes required for ABA biosynthesis and response (Cutler *et al.* 2010). Nuclear ABA perception occurs through the PYRABACTIN RESISTANCE/PYRABACTIN RESISTANCE1-LIKE/REGULATORY COMPONENT OF ABA RECEPTOR 1 (PYR/PYL/RCAR1) family (Ma *et al.* 2009; Park *et al.* 2009). PYR/PYL/RCAR proteins directly bind and regulate protein phosphatase type 2C (PP2C) proteins, such as ABA INSENSITIVE1 (ABI1) and ABI2, which have long been implicated in ABA signaling (Leung *et al.* 1997). Under high ABA concentrations, these PP2C proteins are inactivated by PYR/PYL/RCAR, allowing for the phosphorylation of SUCROSE NONFERMENTING1-RELATED PROTEIN KINASE2 (SnRK2) family members, which phosphorylate downstream ABA signaling components to mediate a response (Nakashima *et al.* 2009). In addition, auxin and ethylene signaling components have been identified as necessary for full ABA responsiveness.

Auxin is an essential plant hormone that regulates plant cell division and elongation (Perrot-Rechenmann 2010) to control nearly every aspect of plant growth and development, including root elongation, embryo patterning, vascularization, cell division and elongation, and many others (Mockaitis and Estelle 2008). Genetic studies in Arabidopsis have uncovered components of the auxin signaling pathway, and much is known about auxin biosynthesis (Korasick *et al.* 2013), transport (Petrášek and Friml 2009), and response (Sauer *et al.* 2013). TRANSPORT INHIBITOR RESPONSE1 (TIR1) and the related AUXIN SIGNALING F-BOX (AFB) family members, which are components of SCF E3 ubiquitin ligases (Parry and Estelle 2006), bind auxin (Dharmasiri *et al.* 2005; Kepinski and Leyser 2005), allowing for interaction with AUXIN/INDOLE-3-ACETIC ACID INDUCIBLE (Aux/IAA) transcriptional repressors. The interaction of SCF^TIR1/AFB^ with Aux/IAA repressor proteins results in the ubiquitylation (Dos Santos Maraschin *et al.* 2009) and subsequent degradation of the multimeric (Korasick *et al.* 2014) Aux/IAA repressors, thus allowing expression of auxin-responsive genes.

Although the roles of ABA and auxin in plant growth are distinct, sensitivity to auxin correlates with sensitivity to ABA. For example, mutations in auxin response genes such as *AUXIN RESISTANT1* (*AUX1)*, *AUXIN RESISTANT1* (*AXR1)*, *IBA RESPONSE5* (*IBR5*), and *TIR1*, which were identified for conferring auxin resistance or resistance to auxin transport inhibitors (Lincoln *et al.* 1990; Bennett *et al.* 1996; Ruegger *et al.* 1998; Monroe-Augustus *et al.* 2003), also confer ABA resistance (Tiryaki and Staswick 2002; Monroe-Augustus *et al.* 2003; Strader *et al.* 2008a). Conversely, the *sax1* mutant displays hypersensitivity to both auxin and ABA (Ephritikhine *et al.* 1999). Interestingly, the ABA-regulated AP2 domain transcription factor *ABI4* was recently shown to repress expression of the auxin transporter *PIN1*, and accordingly *abi4* mutants display enhanced root auxin transport (Shkolnik-Inbar and Bar-Zvi 2011). These connections suggest that auxin responsiveness is required for plants to be able to fully respond to ABA.

Ethylene is a gaseous phytohormone that affects many aspects of plant growth and development, including fruit ripening, senescence, and leaf abscission (Kendrick and Chang 2008). The rate-limiting step in ethylene biosynthesis is controlled by 1-aminocyclopropane-1-carboxylic acid (ACC) synthase (ACS) enzymes (Chae and Kieber 2005). A subset of these enzymes are targeted for degradation by the ETHYLENE OVERPRODUCER1 (ETO1) E3 ubiquitin ligase; mutation of ETO1 results in increased stability of these ACS enzymes and ethylene hyperproduction (Chae and Kieber 2005). Ethylene is perceived by a family of transmembrane histidine kinase receptors, including ETHYLENE RECEPTOR1 (ETR1), ETR2, ETHYLENE RESPONSE SENSOR1 (ERS1), ERS2, and ETHYLENE INSENSITIVE4 (EIN4) (Kendrick and Chang 2008). In the absence of ethylene, these receptors activate the Raf-like protein kinase CONSTITUTIVE TRIPLE RESPONSE1 (CTR1) (Kieber *et al.* 1993), which represses the EIN2 ethylene response activator (Alonso *et al.* 1999). In the presence of ethylene, the ethylene receptors fail to activate CTR1, relieving repression of EIN2 and allowing for EIN2 activation of the EIN3 family of transcription factors (Kendrick and Chang 2008). Many auxin-resistant mutants display ethylene resistance in root elongation. Well-characterized examples include *aux1* (Pickett *et al.* 1990), *ibr5* (Strader *et al.* 2008b), and *tir1* (Alonso *et al.* 2003), suggesting that auxin responsiveness is necessary for full ethylene responsiveness.

ABA and ethylene interactions are complex and not well-characterized. Many ethylene signaling mutants simultaneously display resistance to ABA in root elongation assays and hypersensitivity to ABA in seed germination assays (Gazzarrini and McCourt 2003). In addition, ABA and ethylene appear to interact to regulate plant stress responses. For example, ABA limits ethylene production in water-stressed plants (Sharp 2002; Sharp and Lenoble 2002), whereas ethylene antagonizes stomatal response to ABA under oxidative stress (Wilkinson and Davies 2009). Intriguingly, ABA inhibits transcription of the ethylene biosynthesis gene ACC OXIDASE (ACO) during seed germination in *Lepidium sativum* (Linkies *et al.* 2009) to limit ethylene production, suggesting a mechanism for ABA regulation of ethylene for seed germination regulation. However, the molecular details of ethylene inhibition of seed germination and ABA-responsive root elongation remain largely unknown.

In addition to ABA–auxin and ABA–ethylene interactions, ethylene and auxin display extensive interactions (Stepanova and Alonso 2009; Muday *et al.* 2012). Each hormone stimulates the biosynthesis of the other; ethylene stimulates IAA production (Růžička *et al.* 2007; Swarup *et al.* 2007) and auxin stimulates ethylene production (Tsuchisaka and Theologis 2004). Also, many ethylene signaling mutants are auxin-resistant, and many auxin signaling mutants are ethylene-resistant (Stepanova *et al.* 2007), suggesting that some facets of auxin signaling require ethylene response and some aspects of ethylene signaling require auxin response. Further supporting this connection, biosynthesis mutants with decreased IAA are mildly ethylene-resistant (Stepanova *et al.* 2005; Stepanova *et al.* 2008). From these auxin–ethylene interactions, a model has emerged (Stepanova and Alonso 2009) for ethylene regulation of root elongation in which ethylene promotes auxin biosynthesis in root meristems (Růžička *et al.* 2007; Swarup *et al.* 2007), followed by auxin transport from the meristem to root elongation zones in an ethylene-regulated manner (Růžička *et al.* 2007; Swarup *et al.* 2007) where auxin simultaneously inhibits cell elongation and promotes ethylene responsiveness (Stepanova *et al.* 2007).

To identify factors required for ABA-responsive root elongation, we performed a genetic screen in *Arabidopsis thaliana* for mutants that display ABA resistance in root elongation assays. We characterized 21 mutant isolates in detail and found that 12 of these isolates displayed resistance to ABA in both root elongation and seed germination assays, whereas nine isolates displayed ABA-resistant root elongation and ABA-sensitive seed germination. We used PCR analysis, recombination mapping, and whole genome sequencing to identify mutations in auxin, ethylene, and ABA signaling components defective in seven ABA Root Resistance (AR) mutants. Because we identified auxin and ethylene signaling components in our mutants, we examined AR mutant responses to auxin and ethylene in root elongation assays. We found that many of our isolates also displayed resistance to these hormones. We further characterized ABA responses in known auxin and ethylene mutants and found that all examined auxin and ethylene-resistant mutants displayed resistance to ABA in root elongation, whereas only the auxin-resistant mutants displayed ABA resistance in seed germination assays. Additionally, auxin and ethylene mutants were nonadditive in ABA-responsive root elongation assays, consistent with the possibility that auxin and ethylene act in a linear pathway to regulate ABA-responsive root elongation. Conversely, auxin and ethylene mutants were additive in ABA-responsive seed germination inhibition assays, suggesting that auxin and ethylene act independently to regulate ABA-responsive inhibition of seed germination. Our results illuminate auxin–ethylene interactions regulating ABA response.

## Materials and Methods

### Plant materials and growth conditions

*Arabidopsis thaliana* accessions Colombia (Col-0) and Landsberg *erecta* (L*er*-0) were used as wild-type for experiments, as indicated in the figure legends. Surface-sterilized (Last and Fink 1988) seeds were plated on plant nutrient medium (PN) (Haughn and Somerville 1986), supplemented with 0.5% (w/v) sucrose (PNS), solidified with 0.6% (w/v) agar. The (±)-ABA, IAA, and 2,4-D stocks were dissolved in 100% ethanol and ACC stocks were dissolved in 50% ethanol. Ethanol-supplemented media were used as controls with all treatments normalized to the same ethanol content. Seedlings were grown at 22° under continuous illumination through yellow long-pass filters to decrease indolic compound breakdown (Stasinopoulos and Hangarter 1990) unless otherwise indicated.

### Mutant isolation and nomenclature

*Arabidopsis thaliana* Col-0 seeds were mutagenized with ethyl-methansulfonate (EMS) (Normanly *et al.* 1997). M_2_ seeds were surface-sterilized and plated on Whatman 3M filter paper on top of PNS medium at ∼1500 seeds per 150-mm plate. After 4 d of growth under continuous illumination through yellow long-pass filters, germinated seedlings and filter paper were transferred to PNS supplemented with 10 µM ABA. After an additional 4 d of growth under continuous illumination through yellow long-pass filters, putative ABA root elongation mutants with long roots were selected, transferred to unsupplemented medium to recover, moved to soil, genotyped for the *abi1-1* and *abi2-1* mutations, and allowed to self-fertilize. M_3_ progeny were retested for resistance to ABA in root elongation assays.

### Double mutant isolation

Generation of *eto1-1 ein2-1*, *eto1-1 ibr5-1*, *eto1-1 tir1-1*, *eto1-1 axr1-3*, and *eto1-1 aux1-7* has previously been described (Strader *et al.* 2010). The *ein2-1* (Alonso *et al.* 1999) mutant was crossed to *ibr5-1* (Monroe-Augustus *et al.* 2003), *tir1-1* (Ruegger *et al.* 1998), *axr1-3* (Estelle and Somerville 1987), and *aux1-7* (Maher and Martindale 1980). Double mutants were identified by PCR analysis of F_2_ progeny. Amplification of *EIN2* with EIN2-1 (5*′*-TTCTCCATGCTAACAATCTTCTCCACAGG-3*′*) and the derived cleaved amplified polymorphic sequence primer (Neff *et al.* 2002) EIN2-B*smA*I (5*′*-AGAGTTGGATGTAAAGTACTCTACGTCT-3*′*; altered residue underlined) results in a 186-bp product with one B*smA*I site in *ein2-1* and no sites in wild-type. Amplification of *IBR5* with T1O3.4-1 (5*′*-CCTAATTTCCTCCGTCTGTGAAATCAAGGG-3*′*) and T1O3.4-6 (5*′*-CAAGGCAAAACCCTAACTAAACAAACCG-3*′*) results in a 463-bp product with one A*ci*I site in wild-type and no sites in *ibr5-1*. Amplification of *TIR1* with TIR1-3 (5*′*-TTGAAGAGATAAGGCTGAAGAGGATGG-3*′*) and TIR1-6 (5*′*-AAACCGGAACACGATTATATGGGATGATG-3*′*) results in a 488-bp product with one D*pn*II site in wild-type and two sites in *tir1-1*. Amplification of *AXR1* with AXR1-15 (5*′*-TCTCATATGTACTTTTCCTCGTCCTCTTCAC-3*′*) and the derived cleaved amplified polymorphic sequence primer (Neff *et al.* 2002) AXR1-A*cc*I (5*′*-AAACCAACTTAACGTTTGCATGTCG-3*′*; altered residue underlined) results in a 185-bp product with on A*cc*I site in wild-type and no sites in *axr1-3*. Amplification of *AUX1* with AUX1-3 (5*′*-CATGGGTCAACAAAGCTTTGGATTTTGTCC-3*′*) and AUX1-4 (5*′*-TTCGTGACTTTTACTCCCTTCACGTATACG-3*′*) results in a 464-bp product with two D*pn*II sites in wild-type and three sites in *aux1-7*.

### Phenotypic assays

All phenotypic assays were performed at least three times, and the presented results are representative of these assays. For auxin-responsive and 1-aminocyclopropane-1carboxylic acid (ACC)–responsive root elongation assays, stratified seeds were grown for 8 d under continuous illumination through yellow long-pass filters on the indicated hormone concentrations and primary root lengths were measured. For ABA-responsive root elongation assays, imbibed seeds were incubated at 4′ for 2 d then plated on unsupplemented PNS medium and incubated at 22° under continuous illumination through yellow long-pass filters for 4 d to allow efficient germination. Seedlings then were transferred to medium supplemented with either ethanol or the indicated concentration of ABA and total primary root lengths were measured after an additional 4 d of growth under continuous illumination through yellow long-pass filters.

For ABA-responsive seed germination and seedling development assays, seeds from plants grown simultaneously under continuous light were afterripened at room temperature for 1 to 2 months before assessment of seed germination properties. Sterile, imbibed seeds were incubated at 4° for 2 d in the dark and then plated on PN supplemented with ethanol (Mock) or the indicated concentrations of ABA and grown under white light. Seeds were examined at the indicated time points and seeds with an emerged, elongating radicle were counted.

### Identification of the *abi2-11*, *aux1-53*, *aux1-99*, and *ein2-291* mutations

The *abi2-11* mutation (in the Col-0 background) was identified in isolate AR11 by PCR-based genotyping M_2_ individuals identified in the AR mutant screen. PCR amplification with ABI2-1 (5*′*-ACGGTGAATCTAGGGTTACTTTAC-3*′*) and ABI2-2 (5*′* -ACTCCGGTTTCTCCTTCACTATC-3*′*) results in a 586-bp product with one *Nco*I site in wild-type and no sites in *abi2-1* or *abi2-11*.

The *aux1-53* and *aux1-99* mutations (in the Col-0 background) were identified in isolates AR53 and AR99, respectively, by recombination mapping. AR53 and AR99 were crossed to Landsberg *erecta* and resultant F_2_ progeny were selected for exhibiting a long root on ABA. DNA from ABA-resistant individuals was scored using PCR-based polymorphic markers (Konieczny and Ausubel 1993; Bell and Ecker 1994). The *AUX1* gene within the AR53 and AR99 mapping intervals was PCR-amplified and sequenced from AR53 and AR99 genomic DNA.

The *ein2-291* mutation (in the Col-0 background) was identified in isolate AR291 by recombination mapping. AR291 was crossed to Landsberg *erecta* and resultant F_2_ were selected for exhibiting a long root on ABA. DNA from ABA-resistant individuals was scored using PCR-based polymorphic markers (Konieczny and Ausubel 1993; Bell and Ecker 1994). The *EIN2* gene within the AR291 mapping interval was PCR-amplified and sequenced from AR291 genomic DNA.

### Identification of the *aux1-*116, *axr4-241*, and *eir1-211* mutations by whole genome sequencing

The *aux1-*116, *axr4-241*, and *eir1-211* mutations were identified in isolates AR116, AR241, and AR211, respectively, by whole genome sequencing. AR116 was crossed to wild type (Col-0) and resultant F_2_ progeny were selected for exhibiting a long root on ABA, moved to soil, and allowed to self-fertilize. Resultant F_3_ progeny were retested for ABA resistance in root elongation. Tissues from 500 seedlings from each of seven ABA-resistant F_3_ pools from the AR116 backcross were combined for genomic DNA extraction. Tissue from pooled M_4_ seedlings was used for AR211 and AR241 genomic DNA extractions.

For genomic DNA extraction, tissue was ground with mortar and pestle under liquid nitrogen to a fine powder and transferred to 7 mL extraction buffer (110 mM Tris, pH 8.0; 55 mM ethylenediaminetetraacetic acid, pH 8.0; 1.54 M NaCl, 1.1% cetyl trimethyl ammonium bromide) prewarmed to 65° in a 15-mL polypropylene conical tube. Tubes were capped and vortexed to incorporate ground tissue; 0.7 mL 20% sodium dodecyl sulfate was added to each sample and mixed by inversion. Samples were incubated in a 65° water bath for 2 hr with occasional inversion. Samples were then cooled to room temperature and 24:1 chloroform:isoamyl alcohol was added to the 14-mL mark on the conical tube. Samples were mixed by inversion for 15 min, and then phases were separated by centrifugation for 15 min at 3000 rpm. After centrifugation, the top (aqueous) phase was removed to a new 15-mL polypropylene conical tube and the chloroform:isoamyl extraction was repeated. The top (aqueous) phase from the second extraction was removed to a new 15-mL polypropylene conical tube and 0.6 volumes isopropanol were added. DNA was precipitated by gentle rocking and collected by centrifugation for 5 min at 3000 rpm. The supernatant was discarded and DNA was resuspended in 4 mL TE buffer (100 mM Tris, 10 mM ethylenediaminetetraacetic acid; pH 8.0). RNaseA was added to a final concentration of 10 µg/mL and incubated for 1 hr at 37°. After the RNaseA digest, 8 mL chloroform was added to each sample and mixed by inversion for 10 min, and then phases were separated by centrifugation for 15 min at 3000 rpm. After centrifugation, the top (aqueous) phase was removed to a new 15-mL polypropylene conical tube and 0.1 volume 3 M sodium acetate (pH 5.2) and 2 volumes 95% chilled ethanol added. DNA was precipitated by gentle rocking and collected by centrifugation for 20 min at 2000 rpm. The supernatant was discarded and 3 mL 70% ethanol was placed on top of the precipitated DNA. After 1 min, ethanol was removed and the tube was inverted to drain and dry the DNA pellet. DNA resuspended in 500 µL TE buffer.

Genomic DNA was sequenced using by the Washington University Genome Technology Assistance Center (GTAC; https://gtac.wustl.edu). Libraries were prepared using an Illumina Genomic DNA kit and sequenced on an Illumina HiSequation 2000 using multiplexing in a 100-bp paired end run.

Reads were aligned to the *Arabidopsis thaliana* Col-0 reference genome with Arabidopsis Information Resource 10 gene annotations (The Arabidopsis Genome Initiative 2000) using Novoalign (Novocraft, http://novocraft.com). SNPs were identified using SAMtools (Li *et al.* 2009) and annotated using snpEFF (Cingolani *et al.* 2012) to predict the effects of variants on genes. We then identified homozygous, canonical EMS-induced changes (G-to-A or C-to-T) from each population. Recombination mapping in the backcross using markers based on identified homozygous EMS-induced mutations was used to determine linkage of identified mutations to phenotype in AR241 and AR211 (Supporting Information, Table S1).

## Results

### Isolation of mutants with reduced ABA responsiveness in root elongation

**Figure 1:**
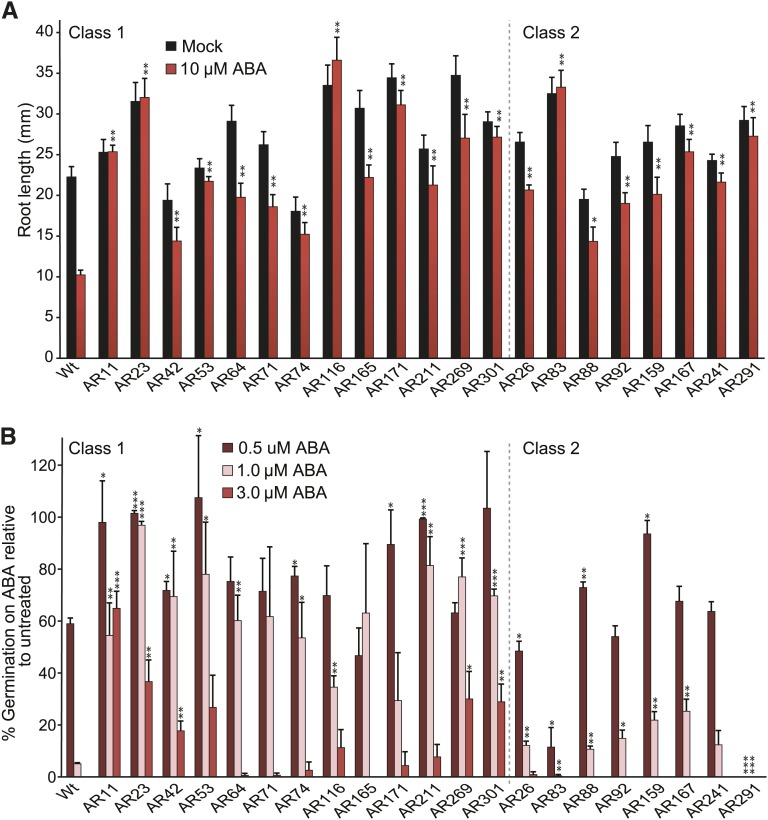
AR mutant responses to ABA in root elongation and seed germination. (A) Mean primary root lengths (±SE; *n* ≥ 15) of Col-0 (Wt) and AR mutant seedlings grown under yellow-filtered light at 22° for 4 d on unsupplemented medium, followed by 4 d on medium supplemented with ethanol (mock) or 10 µM ABA. Asterisks indicate that the mutant root lengths were significantly longer than wild-type root lengths on 10 µM ABA (*P ≤ 0.01; **P ≤ 0.001) in two-tailed *t* tests assuming unequal variance. (B) Mean normalized percentage (±SE; *n* = 3) of Col-0 (Wt) and AR mutant seeds after 5 d grown under yellow-filtered light at 22° on medium supplemented with 0.3 µM ABA, 1.0 µM ABA, or 3 µM ABA. Asterisks indicate that the mutant germination percentages were significantly different from wild-type germination percentages (*P ≤ 0.05; **P ≤ 0.01; ***P ≤ 0.001) in two-tailed *t* tests assuming unequal variance.

ABA inhibits both seed germination and primary root elongation in Arabidopsis (Cutler *et al.* 2010). To isolate mutants displaying ABA-resistant root growth, we generated 56 pools of EMS-mutagenized Col-0 seed and screened approximately 280,000 resultant M_2_ progeny for seedlings with reduced ABA-responsiveness in root elongation. We plated stratified M_2_ seeds on filter paper–lined unsupplemented media and grew them for 4 d to allow for seed germination. We then transferred the newly germinated seedlings atop filter papers to media supplemented with 10 µM ABA. After an additional 4 d of growth, we selected 316 putative mutants from 56 independent pools exhibiting a long root on ABA. Of these, 76 died and eight were infertile. Progeny of the 232 remaining putative mutants were rescreened for resistance to ABA in root elongation; 168 of these mutants displayed longer roots than wild-type in response to ABA. Twenty-one of the ABA-resistant lines were characterized in detail. Because each mutant came from a separate seed pool (Table 1), the mutants represent 21 independent mutagenic events. All 21 isolates demonstrated strong ABA resistance in root elongation assays (Figure 1A), whereas a subset of the isolates failed to demonstrate strong ABA resistance in seed germination (Figure 1B). To classify our mutants, we considered seed germination of more than 25% in the presence of 1 µM ABA after 72 hr of inhibition to be strong ABA resistance. We used these differences in ABA-responsive seed germination to divide our isolates into two mutant classes: class 1 mutants displayed strong resistance to the inhibitory effects of ABA in both root elongation and seed germination, whereas class 2 mutants displayed resistance to the inhibitory effects of ABA on root elongation and hypersensitivity, wild-type sensitivity, or mild resistance to ABA in seed germination inhibition (Table 1). Isolates AR11, AR23, AR42, AR53, AR64, AR71, AR74, AR116, AR165, AR171, AR211, AR269, and AR301 displayed strong ABA resistance in seed germination assays and were placed into class 1. AR26, AR88, AR92, AR159, AR167, and AR241 displayed mild resistance to ABA, and AR83 and AR291 displayed ABA hypersensitivity in seed germination assays and were placed into class 2. Interestingly, the separation of ABA responsiveness in root elongation and seed germination in class 2 mutants is consistent with the possibility that they carry defects in ABA response pathways specific to root length response that are independent from germination response.

**Table 1:** Characterization of mutants

Class	Isolate	M_2_ Pool	Response in Root Elongation Assays			ABA-Responsive Seed Germination*^d^*	Causative Mutation
			ABA*^a^*	ACC*^b^*	*2,4-D**^c^*		
—	Wt	—	••••	••••	••••	••••	
1	AR11	3	•	••••	••••	••	*abi2-11*
1	AR23	6	•	•	•	•	
1	AR42	10	••	••	•••	••	
1	AR53	12	•	•	•	•	*aux1-53*
1	AR64	15	•••	••••	••••	••	
1	AR71	16	••	••••	••••	••	
1	AR74	17	••	••••	••••	••	
1	AR116	27	•	•	•	•••	*aux1-*116
1	AR165	34	••	•••	•	••	
1	AR171	35	•	••••	••••	•••	
1	AR211	42	••	•	••••	•	*eir1-211*
1	AR269	48	••	•	•	•	*iaa16-1*
1	AR301	53	•	•	•	••	
2	AR26	7	••	••••	•••	••••	
2	AR83	19	•	•	•••	•••••	
2	AR88	20	••	••••	••••	••••	
2	AR92	21	••	•••	••••	••••	
2	AR159	33	••	••••	••••	••••	
2	AR167	35	•	••••	••••	••••	
2	AR241	44	•	••••	•••	••••	*axr4-241*
2	AR291	50	•	•	••••	•••••	*ein2-291*
**Known mutants**							
1	*ibr5-1*	—	••	•••	•••	•	*ibr5-1*
1	*tir1-1*	—	•••	•••	•••	•	*tir1-1*
1	*axr1-3*	—	•••	•••	•	•••	*axr1-3*
1	*aux1-7*	—	•	•	•	•	*aux1-7*
2	*ein2-1*	—	•	•	•••	•••••	*ein2-1*
—	*eto1-1*	—	•••	•	••••	•••	*eto1-1*
2	*etr1-1*	—	•	•	•••	S*^e^*	*etr1-1*
1	*abi1-1*	—	•	••••	••••	R*^f^*	*abi1-1*
1	*abi2-1*	—	••	••••	••••	R*^f^*	*abi2-1*
1	*abi3-1*	—	••••	••••	••••	R*^f^*	*abi3-1*

a Relative ABA responsiveness on 10 μM ABA *vs.* mock-treated. •••• = 0–60%; ••• = 60–70%; •• = 71–85%; and • = 86–100%.

b Relative ACC responsiveness on 100 nM ACC *vs.* mock-treated. •••• = 0–70%; ••• = 70–80%; •• = 80–90%; and • = 90–100%.

c Relative 2,4-D responsiveness on 100 nM 2,4-D *vs.* mock-treated. •••• = 0–50%; ••• = 50–65%; •• = 65–85%; and • = 85–100%.

d Relative ABA inhibition of seed germination on 1 μM ABA *vs.* mock-treated. ••••• = 0–5%; •••• = 5–25%; ••• = 25–50%; •• = 50–75%; and • = 75–100%.

e Beaudoin *et al.* (2000). S = Sensitive.

f Koornneef *et al.* (1984). R = Resistant.

### PCR-based genotyping to identify a mutation in *ABI2*

**Figure 2:**
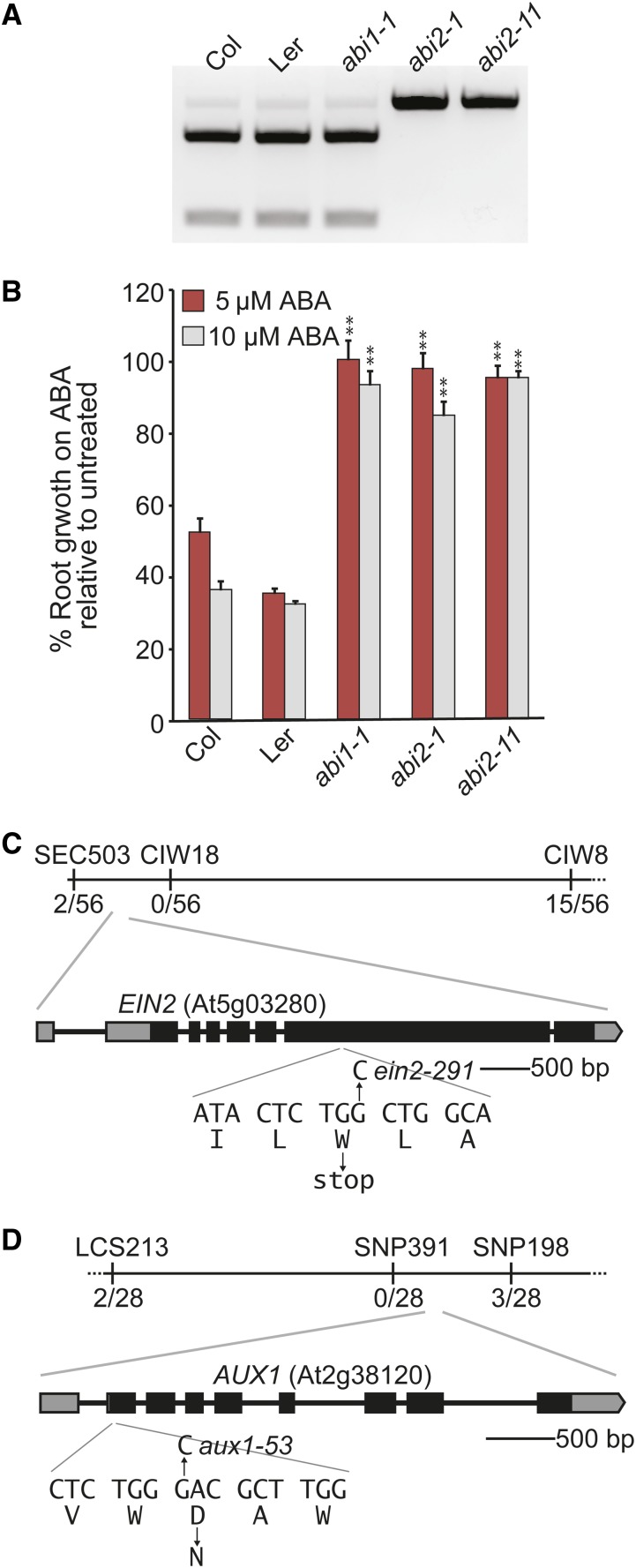
Identification of AR mutant defects in ABA, ethylene, and auxin response factors. (A) Amplification of *ABI2* from wild-type ecotypes Col-0 and L*er*-0, *abi1-1*, *abi2-1*, and *abi2-11*, followed by an N*co*I digest, demonstrates that *abi2-1* and *abi2-11* contain the same mutation that removes the N*co*I restriction site from the sequence. (B) Mean normalized primary root lengths (±SE; *n* = 15) of Col-0, L*er*-0, *abi1-1*, *abi2-1*, and *abi2-11* seedlings grown under yellow-filtered light at 22° for 4 d on unsupplemented medium, followed by 4 d on medium supplemented with 5 or 10 µM ABA compared to seedlings grown on medium supplemented with ethanol (mock). Asterisks indicate that the mutant normalized root lengths were significantly longer than wild-type normalized root lengths (**P ≤ 0.001) in two-tailed *t* tests assuming unequal variance. (C) Recombination mapping with PCR-based markers SEC503, CIW18, and CIW8 localized the ABA resistance mutation in AR291 between SEC503 and CIW8 with 2/56 north and 15/56 south recombinants. Examination of the *EIN2* (*At5g03280*) gene in this region revealed a G-to-A mutation at position 1881 in AR291 DNA that results in Trp455-to-stop. (D) Recombination mapping with PCR-based markers LCS213, SNP391, and SNP198 localized the ABA resistance mutation in AR53 between LCS213 and SNP198 with 2/28 north and 3/28 south recombinants. Examination of the *AUX1* (*At2g38120*) gene in this region revealed a G-to-A mutation at position 148 in AR53 DNA that results in an Asp50-to-Arg substitution.

The gain-of-function *abi1-1* and *abi2-1* mutants display resistance to ABA in root elongation assays (Koornneef *et al.* 1984); therefore, we expected to identify alleles of *abi1-1* and *abi2-1* in our screen. We examined M_2_ isolates for the *abi1-1* and *abi2-1* lesions by PCR-based genotyping. Both *abi1-1* and *abi2-1* alleles result from similar C-to-T transitions that result in the loss of an N*co*I site in *ABI1* and *ABI2* (Leung *et al.* 1997). Although we did not isolate an *abi1-1* allele among our isolates, AR11, a class 1 mutant (Table 1), carried a C-to-T transition in *ABA INSENSITIVE2*/*At5g57050* identical to the *abi2-1* mutant in the L*er* ecotype (Figure 2A). We therefore named the AR11 isolate *abi2-11*. *abi2-11* displayed ABA resistance in root elongation assays similar to *abi1-1* and *abi2-1* (Figure 2B). Because both *abi1-1* and *abi2-1* are in the L*er* ecotype, the *abi2-11* allele in the Col-0 ecotype may be a useful tool for future genetic studies.

### Positional cloning to identify mutations in *AUX1* and *EIN2*

We used ABA resistance in root elongation to map the recessive mutation in AR291 to a region on the upper arm of chromosome 5 between SEC503 and CIW8 (Figure 2C). This region contains *ETHYLENE INSENSITIVE2*/*At5g03280* (*EIN2*). Mutations in *EIN2* have previously been described to display ABA resistance in root elongation and hypersensitivity to ABA in seed germination assays (Beaudoin *et al.* 2000; Ghassemian *et al.* 2000), making a mutation in *EIN2* a reasonable candidate for conferring the ABA resistance found in AR291, which is resistant to ABA in root elongation (Figure 1A) and hypersensitive to ABA in seed germination (Figure 1B) assays. We PCR-amplified and sequenced *EIN2* from AR291 genomic DNA and identified a G-to-A base change at position 1881 (where the A of the ATG is at position 1) that causes a Trp455-to-stop mutation. We named the identified mutation in AR291 *ein2-291*.

We further used ABA resistance in root elongation to map the recessive mutation in AR53 to a region on the lower arm of chromosome 2 between the mapping markers LCS213 and SNP198 (Figure 2D). This region contains *AUXIN RESISTANT1*/*At2g38120* (*AUX1*). Mutations in *AUX1* have previously been described to display ABA resistance in root elongation (Strader *et al.* 2008a) and seed germination (Belin *et al.* 2009) assays, making a mutation in *AUX1* a reasonable candidate for conferring the ABA resistance found in AR53. We PCR-amplified and sequenced *AUX1* from AR53 genomic DNA and identified a G-to-A base change at position 148 (where the A of the ATG is at position 1) that causes a D50-to-N mutation. We named the identified mutation in AR53 *aux1-53*.

### Whole genome sequencing to identify mutations in *AUX1*, *AXR4*, and *EIR1*/*PIN2*

**Figure 3:**
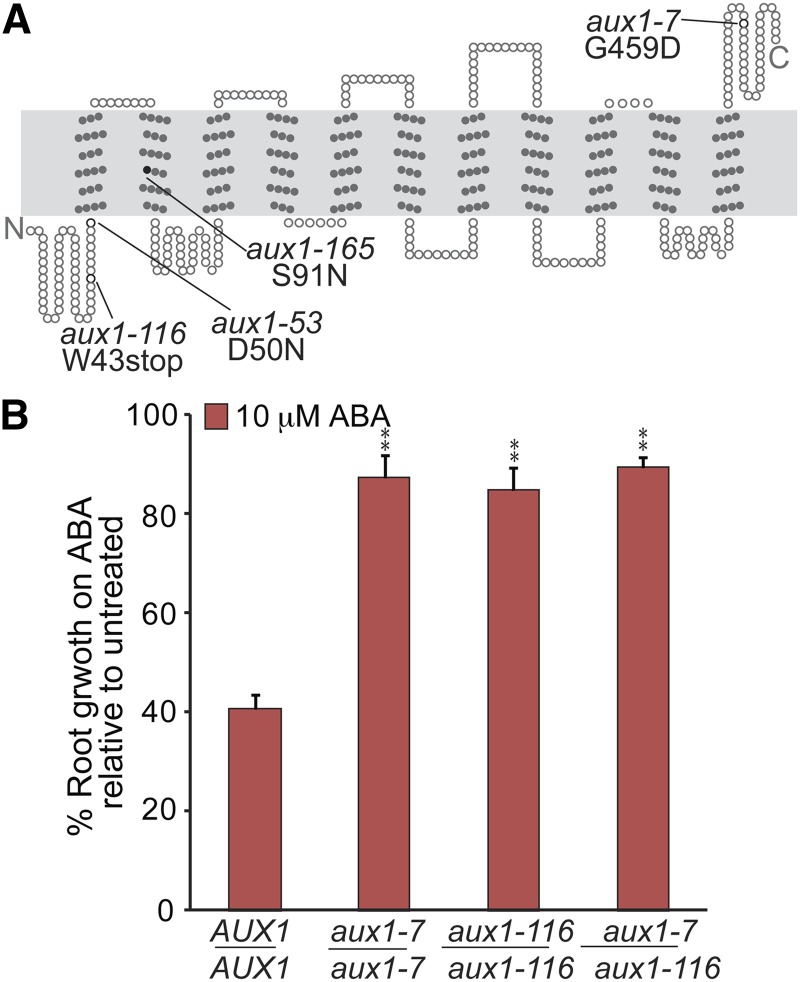
Whole genome sequencing of AR116 uncovered a new allele of *aux1*. (A) Examination of *AUX1* in AR116 revealed a G-to-A mutation in the first exon, which causes a Trp43-to-stop mutation. The AR53 causative mutation (*aux1-53*), identified by recombination mapping (Figure 2D), is also shown. (B) *aux1-116* is allelic to *aux1-7*. Complementation test showing mean normalized primary root length (±SE; *n* ≥ 13) of Col-0 wild-type (*AUX1*/*AUX1*), *aux1-7*/*aux1-7*, *aux1-116*/*aux1-116*, and *aux1-7*/*aux1-116* seedlings grown under yellow-filtered light at 22° for 4 d on unsupplemented medium, followed by 4 d on medium supplemented with 10 µM ABA. Asterisks indicate that the mutant normalized root lengths were significantly longer than wild-type normalized root lengths on ABA (**P ≤ 0.001) in two-tailed *t* tests assuming unequal variance.

We isolated genomic DNA from seven pooled backcrossed lines (bulk segregants) of AR116 and used whole genome sequencing to quickly identify the potential causative EMS mutation. Because EMS typically causes G-to-A or C-to-T base pair changes, and because we assumed the causative mutation would be homozygous in the population, we mined the sequencing data for homozygous G-to-A or C-to-T base pair changes and found a total of 8 homozygous, EMS-related mutations not present in our wild-type sequence that clustered on the long arm of chromosome 2 (Table 2). This region included a promising candidate gene, *AUXI*/*At2g38120*, encoding an auxin influx carrier (Bennett *et al.* 1996; Marchant *et al.* 1999) that is necessary for ABA-responsive root elongation (Strader *et al.* 2008a). The EMS-generated mutation in *AUX1* causes a G-to-A base pair change in the first exon of the coding sequence, resulting in a tryptophan-to-stop mutation at amino acid 43 of the protein (Figure 3A). We crossed AR116 to *aux1-7* and found that the F_1_ progeny displayed resistance to ABA in root elongation assays similar to the two parents (Figure 3B), confirming that AR116 ABA resistance phenotypes are caused by the mutation identified in *AUX1*. We named this allele *aux1-*116.

**Table 2:** Homozygous EMS-related mutations in the AR116 exome

Chromosome	Location*^a^*	Reference Sequence	Mutant Sequence	Gene	Amino Acid Change	Codon Change	Annotated Gene
2	14625394	G	A	At2g34680	L/L	ctC/ctT	AIR9
2	15000961	G	A	At2g35690	G/E	gGa/gAa	ACX5
2	15674636	G	A	At2g37360	P/L	cCa/cTa	ABCG2
2	15674636	G	A	At2g37362	W/stop	tGg/tAg	Potential natural antisense gene, locus overlaps with AT2G37360
2	15973621	G	A	At2g38120	W/stop	tgG/tgA	AUX1
2	16725401	G	A	At2g40050	N/N	aaC/aaT	Cysteine/histidine-rich C1 domain family protein
2	17850781	G	A	At2g42890	R/K	aGa/aAa	AML2
2	19007638	G	A	At2g46290	S/S	tcC/tcT	Transducin/WD40 repeat-like superfamily protein

a Base pair location on chromosome 2 in TAIR v10.

**Figure 4:**
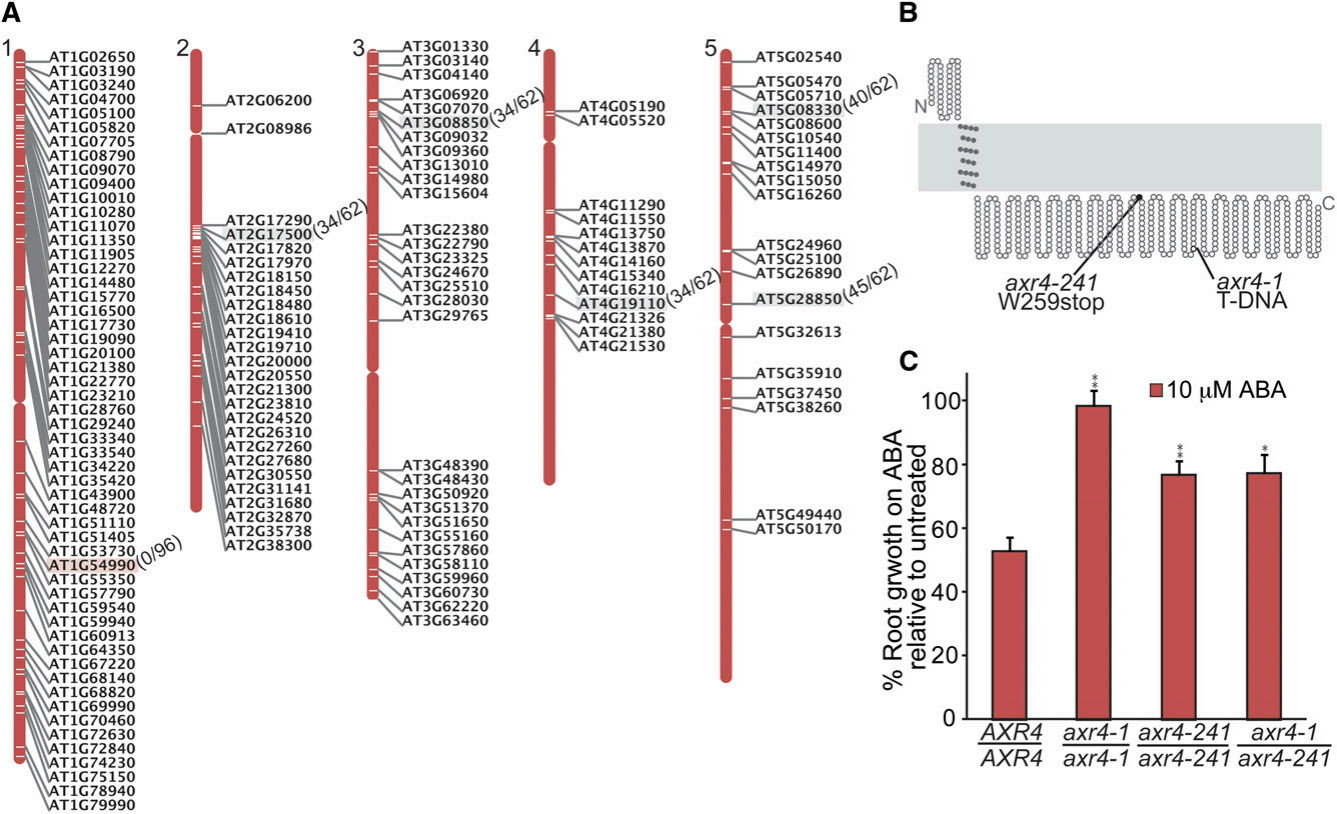
Whole genome sequencing of AR241 revealed a new allele of *axr4*. (A) Map positions of homozygous EMS-caused mutations identified in AR241. Genomic DNA from pooled M_4_ seedlings was sequenced and examined for G/C to A/T transitions typically associated with EMS mutagenesis in splice sites and coding sequences (see Table S1 for list of mutations identified). Approximate map positions of the identified mutations are shown to the right of each chromosome. PCR-based markers (Table S2) were designed for a subset of identified mutations and used to localize the AR241 causative mutation near *At1g54990* with 0/96 recombination events in a population of AR241 backcrossed lines. Recombination events in examined backcrossed lines are listed to the right of gene names for each PCR-based marker. (B) Examination of *AXR4*/*At1g54990* in AR241 revealed a G-to-A mutation in the first exon causing a Trp259-to-stop mutation. (C) *axr4-241* is allelic to *axr4-1*. Complementation test showing mean normalized primary root lengths (±SE; *n* ≥ 10) of Col-0 wild-type (*AXR4*/*AXR4*), *axr4-1*/*axr4-1*, *axr4-241*/*axr4-241*, and *axr4-1*/*axr4-241* seedlings grown under yellow-filtered light at 22° for 4 d on unsupplemented medium, followed by 4 d on medium supplemented with 10 µM ABA. Asterisks indicate that the mutant normalized root lengths were significantly longer than wild-type normalized root lengths on ABA (*P ≤ 0.01; **P ≤ 0.001) in two-tailed *t* tests assuming unequal variance.

To identify the causative mutation in AR241, we isolated genomic DNA from pooled M_4_ seedlings and used whole genome sequencing to quickly identify the potential causative EMS mutation. Because this mutant had not been backcrossed to reduce the number of background mutations, our sequencing data revealed 144 homozygous EMS-related mutations not present in our wild-type sequence that were found throughout the genome (Figure 4A, Table S1). We then crossed AR241 to Col-0 and F_2_ progeny were selected for the ABA root elongation resistance phenotype. CAPS-based and dCAPS-based PCR genotyping primers were designed for identified mutations (Table S2) to determine linkage between ABA-resistant root elongation and the identified mutations. Genotyping for the *AUXIN RESISTANT4*/*At1g54990* (*AXR4*) (Dharmasiri *et al.* 2006) mutation revealed that all of the individuals tested were homozygous at the *AXR4* locus, suggesting this may be the locus responsible for the AR241 phenotype. The EMS-generated mutation in *AXR4* causes a G-to-A base pair change in the first exon of the coding sequence, resulting in a tryptophan-to-stop mutation at amino acid 259 of the protein (Figure 4B). We crossed AR241 to *axr4-1* (Hobbie and Estelle 1995) and found that the F_1_ progeny displayed resistance to ABA in root elongation assays similar to the two parents (Figure 4C), confirming that AR241 ABA resistance phenotypes are caused by the mutation identified in *AXR4*, and we named this new allele *axr4-241*.

**Figure 5:**
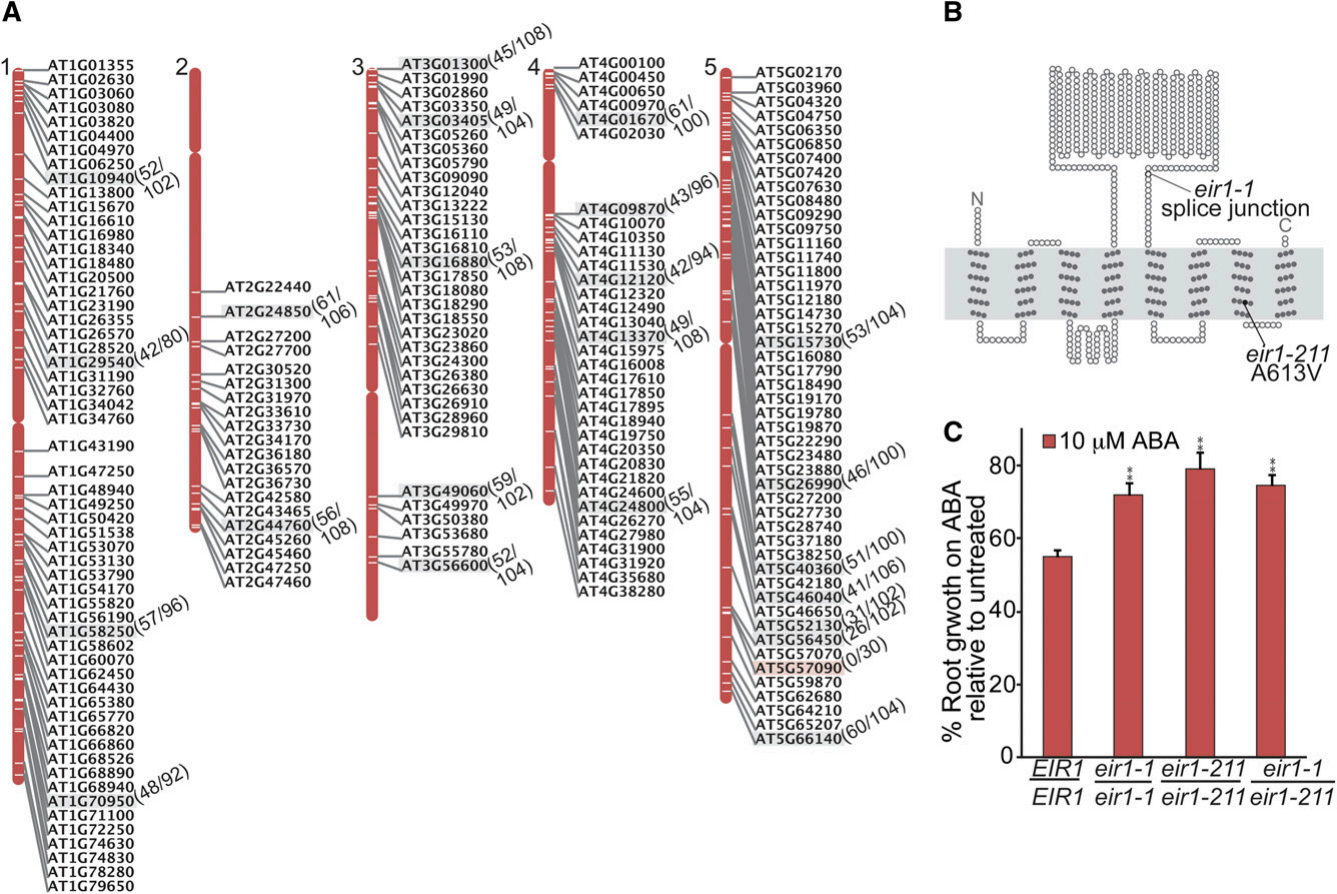
Whole genome sequencing of AR211 reveals a new allele of *eir1*. (A) Map positions of homozygous EMS-caused mutations identified in AR211. Genomic DNA from pooled M_4_ seedlings was sequenced and examined for G/C to A/T transitions typically associated with EMS mutagenesis in splice sites and coding sequences (see Table S3 for list of mutations identified). Approximate map positions of the identified mutations are shown to the right of each chromosome. PCR-based markers (Table S2) were designed for a subset of identified mutations and used to localize the AR211 causative mutation near *At5g57090* with 0/30 recombination events in a population of AR211 backcrossed lines. Recombination events in examined backcrossed lines are listed to the right of gene names for each PCR-based marker. (B) Examination of *EIR1/PIN2* in AR211 revealed a C-to-T mutation in the eighth exon causing an Ala613-to-Val mutation. (C) *eir1-211* is allelic to *eir1-1*. Complementation test showing mean normalized primary root lengths (±SE; *n* = 15) of Col-0 wild-type (*EIR1*/*EIR1*), *eir1-1*/*eir1-1*, *eir1-211*/*eir1-211*, and *eir1-1*/*eir1-211* seedlings grown under yellow-filtered light at 22° for 4 d on unsupplemented medium, followed by 4 d on medium supplemented with 10 µM ABA compared to mock-treated (ethanol) seedlings. Asterisks indicate that the mutant normalized root lengths were significantly longer than wild-type normalized root lengths on ABA (**P ≤ 0.001) in two-tailed *t* tests assuming unequal variance.

To identify the causative mutation in AR211, we isolated genomic DNA from pooled M_4_ seedlings and used whole genome sequencing to quickly identify the potential causative EMS mutation. Because this mutant had not been backcrossed to reduce the number of background mutations, our sequencing data revealed 195 homozygous EMS-related mutations not present in our wild-type sequence that were found throughout the genome (Figure 5, Table S3). We then crossed AR211 to Col-0 and selected F_2_ progeny for the ABA root elongation resistance phenotype. CAPS-based and dCAPS-based PCR genotyping primers (Table S2) were designed to screen these seedlings for the presence of the EMS mutations identified in the genome sequencing. Genotyping for the *ETHYLENE INSENSITIVE ROOT1*/*PIN-FORMED 2*/*At5g57090* (*EIR1*/*PIN2*) (Luschnig *et al.* 1998) mutation revealed that all of the individuals tested were homozygous at the *EIR1* locus, suggesting this may be the locus responsible for the AR211 phenotype. The EMS-generated mutation in *EIR1* causes a C-to-T base pair change in the eighth exon of the coding sequence, resulting in an alanine-to-valine missense mutation at amino acid 613 of the protein (Figure 5B). We crossed AR211 to *eir1-1* and found that the F_1_ progeny displayed resistance to ABA in root elongation assays similar to the two parents (Figure 5C), confirming that AR211 ABA resistance phenotypes are caused by the mutation identified in *EIR1/PIN2*, and we named this new allele *eir1-211*.

### AR mutants display varied responses to ACC and auxin

**Figure 6:**
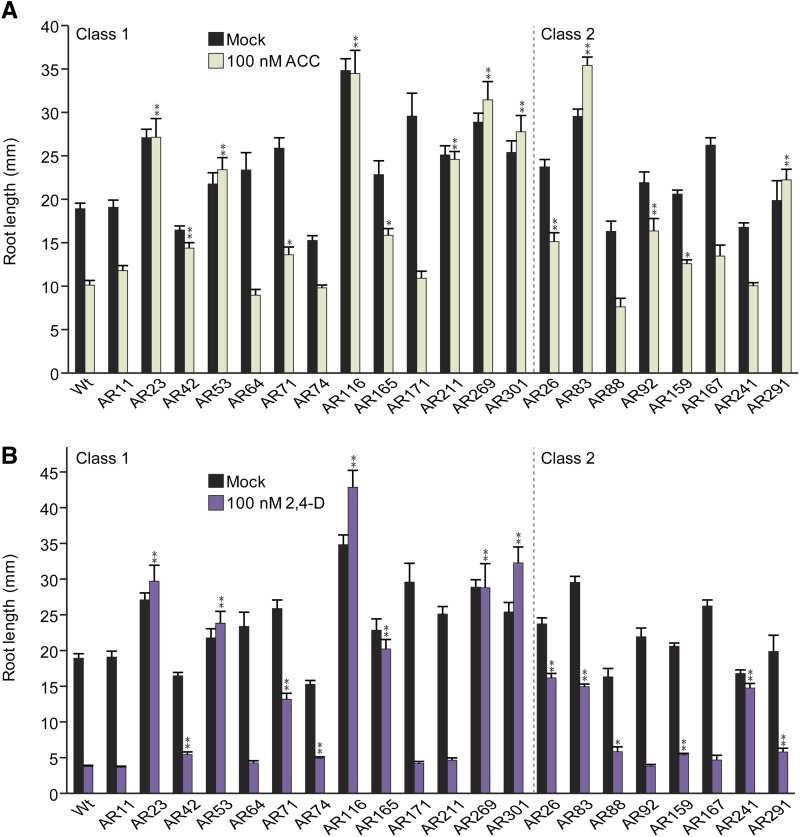
AR mutant responses to ACC and 2,4-D. (A) Mean primary root lengths (±SE; *n* ≥ 12) of 8-day-old Col-0 (Wt) and AR mutant seedlings grown under yellow-filtered light at 22° on medium supplemented with ethanol (mock) or 100 nM ACC. Asterisks indicate that the mutant normalized root lengths were significantly longer than wild-type normalized root lengths on ACC (*P ≤ 0.01; **P ≤ 0.001) in two-tailed *t* tests assuming unequal variance. (B) Mean primary root lengths (±SE; *n* ≥ 12) of 8-day-old Col-0 (Wt) and AR mutant seedlings grown under yellow-filtered light at 22° on medium supplemented with ethanol (mock) or 100 nM 2,4-D. Asterisks indicate that the mutant normalized root lengths were significantly longer than wild-type normalized root lengths on 2,4-D (*P ≤ 0.01; **P ≤ 0.001) in two-tailed *t* tests assuming unequal variance.

Because the causative mutations initially found in the AR isolates disrupted known components of ABA, ethylene, and auxin pathways, we examined AR mutant responsiveness to ethylene and auxin in root elongation assays to determine if the remaining isolates might also be defective in ethylene or auxin signaling. Primary root lengths of the selected AR mutants were measured in response to the ethylene precursor ACC (Figure 6A) and the synthetic auxin 2,4-D (Figure 6B).

Class 1 AR mutants could be subdivided into categories based on hormone responses in root elongation. The class 1 isolates AR11 (defective in *ABI2*), AR64, and AR171 displayed wild-type sensitivity to ACC (Figure 6A) and 2,4-D (Figure 6B) in root elongation assays, consistent with roles for the defective genes in AR11 (*abi2-11*), AR64, and AR171 specifically in ABA response. The class 1 isolate AR53, defective in the auxin transporter AUX1, displayed resistance to ACC (Figure 6A) and 2,4-D (Figure 6B), consistent with previous results suggesting that AUX1 activity is necessary for response to these hormones in root elongation assays (Pickett *et al.* 1990). Likewise, the class 1 isolates AR23, AR42, AR116, AR165, AR269 (defective in IAA16) (Rinaldi *et al.* 2012), and AR301 displayed resistance to ACC (Figure 6A) and 2,4-D (Figure 6B), consistent with the possibility that they may also be defective in components of auxin response.

Class 2 AR mutants could be subdivided into categories based on hormone responses in root elongation assays. The class 2 isolate AR291 (defective in *EIN2*) displayed resistance to ACC (Figure 6A) (Romano *et al.* 1995; Fujita and Syono 1996; Alonso *et al.* 1999) and hypersensitivity to ABA in seed germination assays (Figure 1B). Similarly, the class 2 isolate AR83 displayed strong resistance to ACC (Figure 6A) and hypersensitivity to ABA in seed germination assays (Figure 1B), suggesting that this isolate may also be defective in a component of the ethylene response pathway. Intriguingly, isolates AR88 and AR167, both of which display strong ABA resistance in root elongation (Figure 1A), display wild-type sensitivity to ABA in seed germination assays (Figure 1B) and wild-type sensitivity to ACC (Figure 6A) and 2,4-D (Figure 6B) in root elongation assays, consistent with the possibility that these mutants are defective in components of the ABA response pathway specific to root responses.

### Auxin and ethylene mutants display altered ABA-responsive root elongation

**Figure 7:**
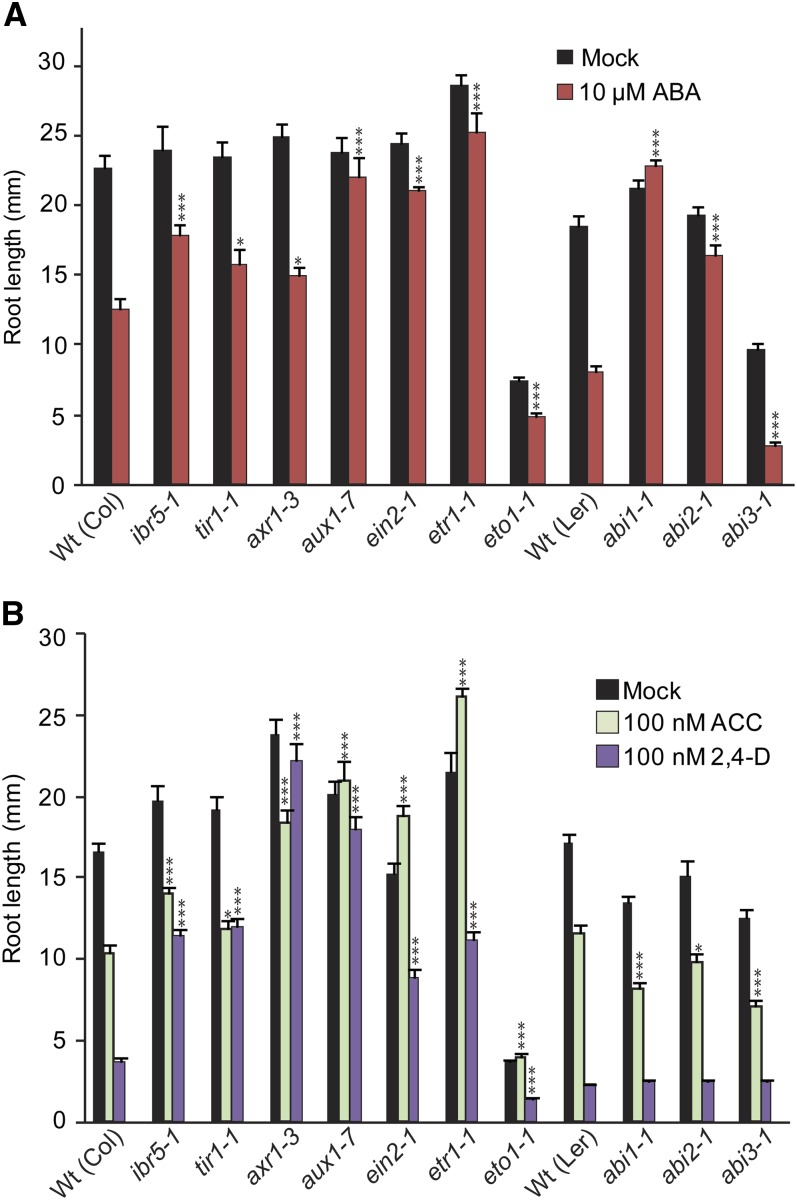
Responses of previously characterized auxin, ethylene, and ABA mutants to hormone treatment. (A) Mean primary root lengths (±SE; *n* = 16) of Col-0, *ibr5-1*, *tir1-1*, *axr1-3*, *aux1-7*, *ein2-1*, *eto1-1*, *etr1-1*, L*er*-0, *abi1-1*, *abi2-1*, and *abi3-1* seedlings grown under yellow-filtered light at 22° for 4 d on unsupplemented medium, followed by 4 d on medium supplemented with ethanol (mock) or 10 µM ABA. Asterisks indicate that the mutant root lengths were significantly different than wild-type root lengths on ABA (*P ≤ 0.05; ***P ≤ 0.001) in two-tailed *t* tests assuming unequal variance. (B) Mean primary root lengths (±SE; *n* = 16) of 8-day-old Col-0, *ibr5-1*, *tir1-1*, *axr1-3*, *aux1-7*, *ein2-1*, *eto1-1*, *etr1-1*, L*er*-0, *abi1-1*, *abi2-1*, and *abi3-1* seedlings grown under yellow-filtered light at 22° on medium supplemented with ethanol (mock), 100 nM ACC, or 100 nM 2,4-D. Asterisks indicate that the mutant root lengths were significantly different than wild-type root lengths on ABA (*P ≤ 0.05; ** *P* ≤ 0.01; *** *P* ≤ 0.001) in two-tailed *t* tests assuming unequal variance.

Because we identified AR mutants with defects in components of auxin, ethylene, and ABA response, we examined several known auxin, ethylene, and ABA mutants for comparison to our unknown isolates. We examined hormone responses of the protein phosphatase mutant *ibr5-1* (Monroe-Augustus *et al.* 2003), the auxin co-receptor mutant *tir1-1* (Ruegger *et al.* 1998), RUB activation mutant *axr1-3* (Lincoln *et al.* 1990), the auxin transport mutant *aux1-7* (Pickett *et al.* 1990), the ethylene response mutant *ein2-1* (Alonso *et al.* 1999), the ethylene overproducing mutant *eto1-1* (Guzmán and Ecker 1990), and the ethylene receptor mutant *etr1-1* (Bleeker *et al.* 1988), all in the Col-0 ecotype, and compared their responses to the hormone responses of the protein phosphatase mutant *abi1-1* (Finkelstein 1994), the protein phosphatase mutant *abi2-1* (Finkelstein 1994), and the ABA transcription factor mutant *abi3-1* (Giraudat *et al.* 1992), all in the L*er*-0 ecotype. We found that, similar to previous reports, the auxin-resistant mutants *ibr5-1* (Monroe-Augustus *et al.* 2003), *tir1-1* (Strader *et al.* 2008a), *axr1-3* (Monroe-Augustus *et al.* 2003), and *aux1-7* (Strader *et al.* 2008a) exhibited ABA-resistant root growth (Figure 7A), consistent with a requirement for intact auxin responsiveness for ABA-responsive inhibition of root elongation. We also found that, similar to previous reports, the ethylene-resistant mutants *ein2-1* (Beaudoin *et al.* 2000) and *etr1-1* (Beaudoin *et al.* 2000) displayed ABA-resistant root growth, suggesting that intact ethylene responses are also required for ABA-responsive inhibition of root elongation.

To complete our characterization, we examined root elongation responses of *ibr5-1*, *tir1-1*, *axr1-3*, *aux1-7*, *ein2-1*, *etr1-1*, *eto1-1*, *abi1-1*, *abi2-1*, and *abi3-1* to the ethylene precursor ACC and the synthetic auxin 2,4-D. As expected, the examined auxin mutants *ibr5-1*, *tir1-1*, *axr1-3*, and *aux1-7* displayed resistance to 2,4-D (Figure 4B). In addition, the ethylene-resistant mutants *ein2-1* and *etr1-1* displayed resistance to 2,4-D (Figure 7B), consistent with previous reports that intact ethylene responses are necessary for full response to exogenous auxins in root elongation assays (Fujita and Syono 1996; Růžička *et al.* 2007; Stepanova *et al.* 2007). Also consistent with previous reports, we found that *ibr5-1* (Strader *et al.* 2008b), *tir1-1* (Alonso *et al.* 2003), *axr1-3* (Timpte *et al.* 1995), and *aux1-7* (Pickett *et al.* 1990) displayed resistance to the ethylene precursor ACC (Figure 7B). *ein2-1* and *etr1-1* displayed strong resistance to ACC and 2,4-D (Figure 7B), consistent with previous reports that mutation of *EIN2* results in resistance to ACC (Alonso *et al.* 1999; Stepanova *et al.* 2007) and the auxin transport inhibitor 1-N-naphthylphthalamic acid (Fujita and Syono 1996), and that *etr1-1* displays resistance to ACC (Chang *et al.* 1993; Beaudoin *et al.* 2000; Růžička *et al.* 2007). Conversely, the ABA signaling mutants *abi1-1*, *abi2-1*, and *abi3-1* displayed wild-type responses in root elongation to the tested concentrations of 2,4-D (Figure 7B), suggesting that ABA responsiveness does not affect auxin responsiveness in root elongation assays. Intriguingly, *abi1-1*, *abi2-1*, and *abi3-1* displayed mild hypersensitivity to the inhibitory effects of ACC on root elongation (Figure 7).

### Auxin and ethylene mutants exhibit altered ABA response in seed germination

**Figure 8:**
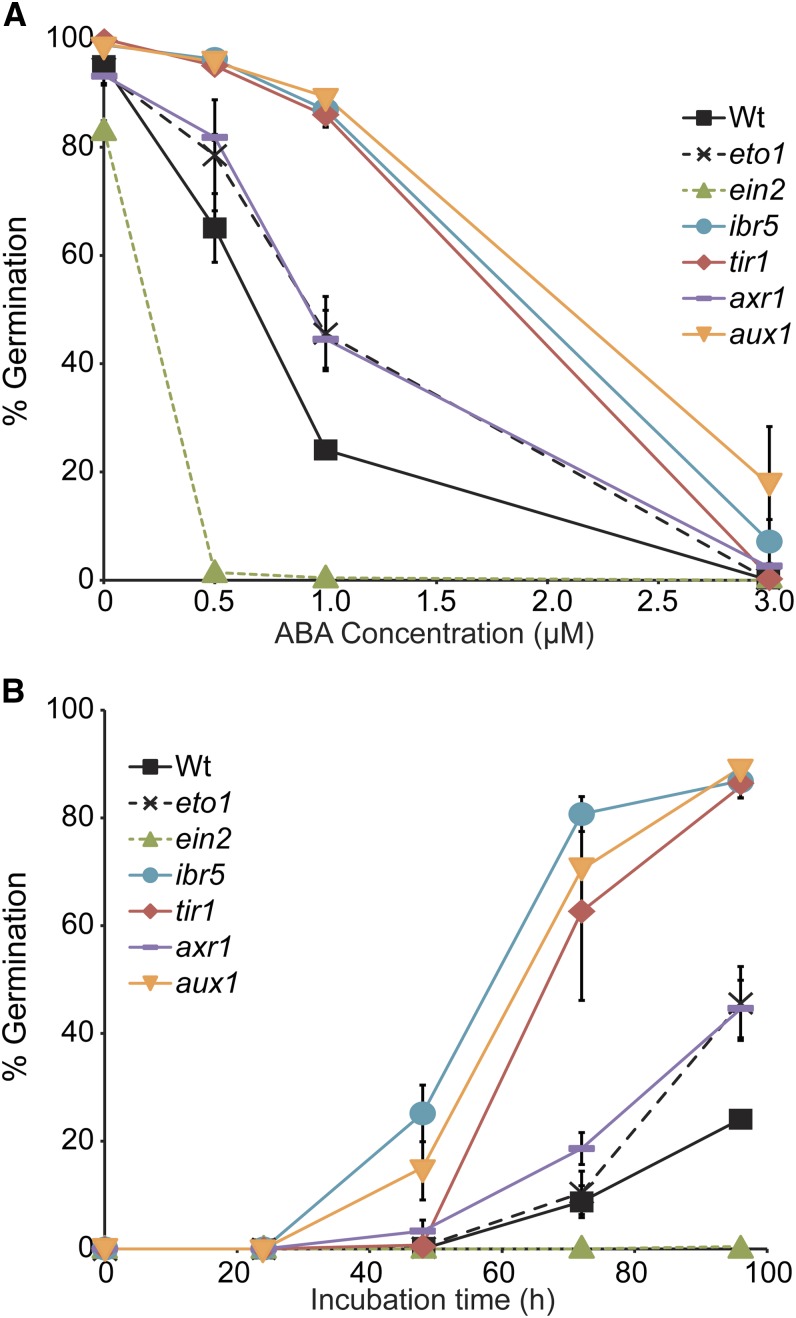
Responses of previously characterized auxin and ethylene mutants to the inhibitory effects of ABA on seed germination. (A) Percentage (±SE; *n* = 3) of Col-0 (Wt), *eto1-1*, *ein2-1*, *ibr5-1*, *tir1-1*, *axr1-3*, and *aux1-7* seed germination on medium supplemented with the indicated concentrations of ABA after 96 hr of growth under white light. *eto1-1*, *ein2-1*, *ibr5-1*, *tir1-1*, *axr1-3*, and *aux1-7* seed germination percentages on 1 µM ABA were significantly different (*P* ≤ 0.02) from Wt seed germination percentage on 1 µM ABA. (B) Percentage (±SE; *n* = 3) of Col-0 (Wt), *eto1-1*, *ein2-1*, *ibr5-1*, *tir1-1*, *axr1-3*, and *aux1-7* seed germination on medium supplemented with 1 µM ABA after the indicated time of growth under white light. *eto1-1*, *ein2-1*, *ibr5-1*, *tir1-1*, *axr1-3*, and *aux1-7* seed germination percentages were significantly different (*P* ≤ 0.02) from Wt seed germination after 96 hr of incubation.

Because our AR mutants could be separated into two classes based on ABA-responsive seed germination, and because the examined auxin and ethylene mutants displayed ABA-resistant root elongation, we examined the auxin and ethylene mutants for altered ABA-responsive seed germination. We found that the auxin mutants *ibr5-1*, *tir1-1*, and *aux1-7* demonstrated strong resistance to ABA inhibition of germination (Figure 8), consistent with previous reports (Monroe-Augustus *et al.* 2003; Belin *et al.* 2009). Conversely, the ethylene-resistant mutant *ein2-1* demonstrated enhanced sensitivity to ABA in seed germination assays (Figure 8), consistent with previous reports that reduced ethylene sensitivity results in increased ABA sensitivity in seed germination assays (Beaudoin *et al.* 2000; Ghassemian *et al.* 2000). These results suggest that ABA-responsive seed germination can be an efficient means of separating ethylene response mutants and auxin response mutants phenotypically.

### Effects of combining auxin and ethylene mutations on ABA responsiveness

**Figure 9:**
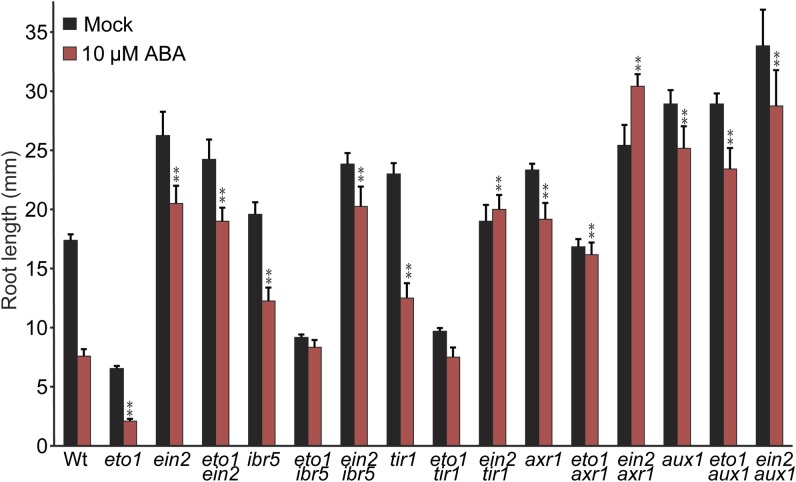
Auxin–ethylene double mutant analysis of ABA-responsive inhibition of primary root elongation. Mean primary root lengths (±SE; *n* ≥ 12) of Col-0, *eto1*, *ein2*, *eto1 ein2*, *ibr5*, *eto1 ibr5*, *ein2 ibr5*, *tir1*, *eto1 tir1*, *ein2 tir1*, *axr1*, *eto1 axr1*, *aux1*, *eto1 aux1*, and *ein2 aux1* seedlings grown under yellow-filtered light at 22° for 4 d on unsupplemented medium, followed by 4 d on medium supplemented with ethanol (mock) or 10 µM ABA. *eto1 ibr5*, *eto1 tir1*, *eto1 axr1*, and *eto1 aux1* root lengths were significantly (*P* ≤ 0.001) longer than the *eto1* root length on ABA. *ein2 ibr5*, *ein2 tir1*, and *ein2 aux1* root lengths were statistically indistinguishable from the *ein2* root length on ABA, whereas *ein2 axr1* root lengths were significantly longer than either *ein2* or *axr1* root lengths (*P* ≤ 0.001) on ABA.

**Figure 10:**
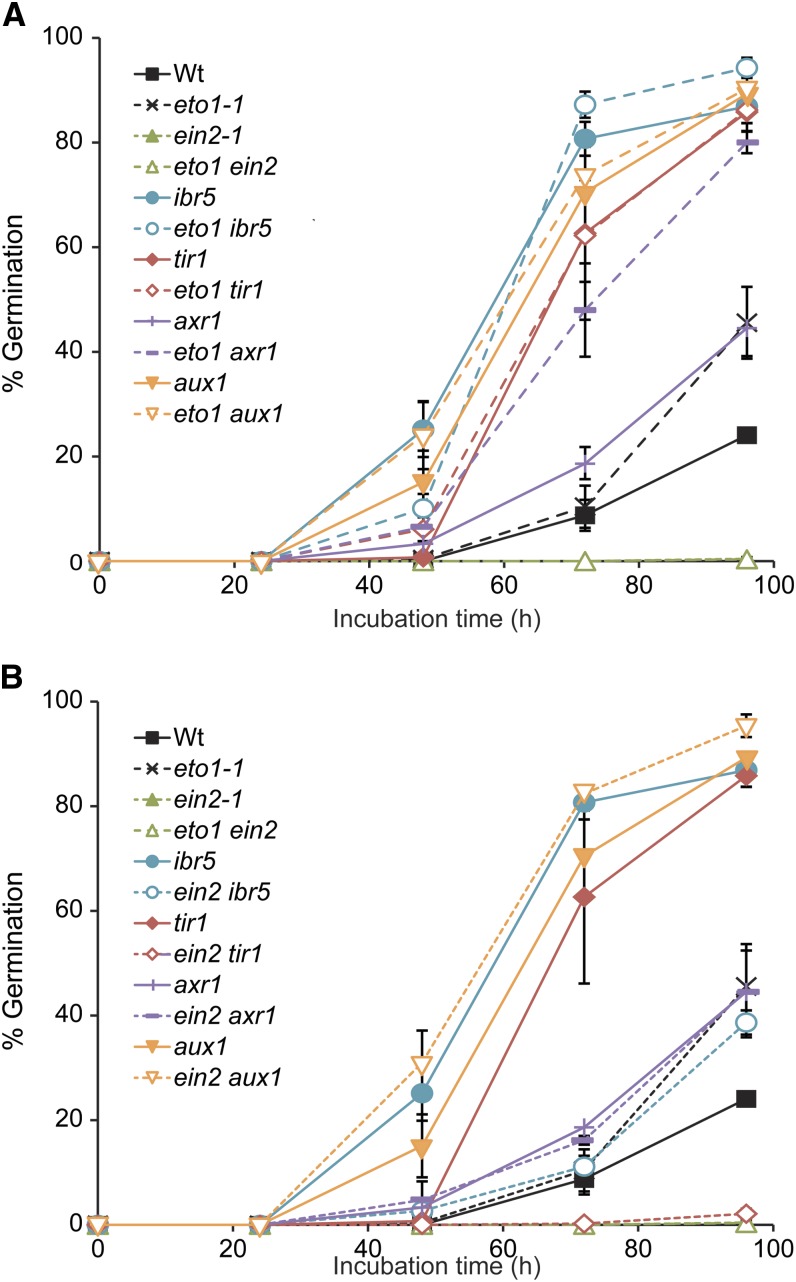
Auxin–ethylene double mutant analysis of ABA-responsive inhibition of seed germination. (A) Percentage seed germination (±SE; *n* = 3) at the indicated time points of Col-0 (Wt), *eto1-1*, *ein2-1*, *eto1-1 ein2-1*, *ibr5*, *eto1 ibr5*, *tir1-1*, *eto1 tir1*, *axr1*, *eto1 axr1*, *aux1*, and *eto1 aux1* seeds grown on medium supplemented with 1 µM ABA. (B) Percentage seed germination (±SE; *n* = 3) at the indicated time points of Col-0 (Wt), *eto1-1*, *ein2-1*, *eto1-1 ein2-1*, *ibr5*, *ein2 ibr5*, *tir1-1*, *ein2 tir1*, *axr1*, *ein2 axr1*, *aux1*, and *ein2 aux1* seeds grown on medium supplemented with 1 µM ABA under white light. *ein2 ibr5*, *ein2 axr1*, and *ein2 aux1* germination percentages were significantly longer than the *ein2* germination percentage (*P* ≤ 0.01) on ABA after 96 hr of incubation.

To further our understanding of the crosstalk between auxin and ethylene response in altering ABA responsiveness, we examined *eto1-1* and *ein2-1* double mutant combinations with the auxin-resistant mutants *ibr5-1*, *tir1-1*, *axr1-3*, and *aux1-7*. For these studies, we used the previously described (Strader *et al.* 2010) double mutants of the ethylene overproducing mutant *eto1-1* (Guzmán and Ecker 1990) with *ibr5-1* (Monroe-Augustus *et al.* 2003), *tir1-1* (Ruegger *et al.* 1998), *axr1-3* (Estelle and Somerville 1987), and *aux1-7* (Maher and Martindale 1980). To generate double mutants with the strong ethylene-resistant mutant *ein2-1* (Alonso *et al.* 1999), we crossed *ein2-1* to *ibr5-1*, *tir1-1*, *axr1-3*, and *aux1-7* and identified double mutants using PCR-based polymorphic markers. In addition, we characterized the *eto1-1 ein2-1* double mutant (Strader *et al.* 2010) to verify whether phenotypes observed in *eto1-1* are caused by increased ethylene signaling rather than altered ACC levels in the *eto1-1* mutant (Figure 9 and Figure 10), which may contribute to ethylene-independent signaling events (Tsang *et al.* 2011).

We examined ABA-responsive root elongation phenotypes of *eto1-1* and *ein2-1* combined with the auxin-resistant mutants *ibr5-1*, *tir1-1*, *axr1-3*, and *aux1-7*. *eto1-1* did not suppress the ABA-resistant root elongation of *ein2-1*, *ibr5-1*, *tir1-1*, *axr1-3*, or *aux1-7* (Figure 9), suggesting that ethylene overproduction cannot compensate for reduced auxin responsiveness in modulating ABA-responsive root elongation. In addition, the *ein2 ibr5*, *ein2 tir1*, and *ein2 aux1* double mutants display ABA-resistant root elongation similar to the *ein2* parent (Figure 9), suggesting that ethylene and auxin response may act linearly to modulate ABA-responsive root elongation. The *ein2 axr1* double mutant displays greater ABA-resistant root elongation than either parent, suggesting *EIN2* and *AXR1* may contribute to ABA responsiveness in root elongation using at least partially distinct mechanisms, consistent with the pleiotropic nature of the *axr1* mutant (Leyser *et al.* 1993), which potentially affects the activity of hundreds of SCF complexes. Results from examining ABA-responsive root elongation phenotypes of these ethylene–auxin double mutants are consistent with auxin and ethylene acting in a linear pathway to affect ABA-responsive inhibition of primary root elongation. Further, because ethylene overproduction by *eto1* could not compensate for the reduced ABA sensitivity in auxin-resistant mutants (Figure 9), auxin is likely downstream of ethylene in this response pathway.

In addition to inhibiting root elongation, ABA inhibits seed germination. Interestingly, auxin and ethylene have been reported to have opposite effects on ABA-responsive inhibition of seed germination and seedling development; auxin-resistant mutants display resistance to ABA in seed germination (Monroe-Augustus *et al.* 2003; Belin *et al.* 2009; Rinaldi *et al.* 2012), whereas ethylene-resistant mutants display hypersensitivity to ABA in seed germination (Subramani 1992; Beaudoin *et al.* 2000; Ghassemian *et al.* 2000). We therefore examined ABA responsiveness in seed germination (Figure 10) assays in our mutants. The *eto1-1* mutant, which overproduces ethylene, displayed mild resistance to the inhibitory effects of ABA on seed germination (Figure 8 and Figure 10A). In addition, *eto1-1* mildly enhanced the ABA resistance in seed germination of the auxin-resistant mutants *ibr5-1*, *axr1-3*, and *aux1-7* (Figure 10A). Conversely, the *ein2-1* mutant, defective in ethylene response, displayed hypersensitivity to the inhibitory effects of ABA on seed germination (Figure 8 and Figure 10B) (Beaudoin *et al.* 2000; Ghassemian *et al.* 2000). *ein2* partially to fully suppressed the ABA resistance in seed germination displayed by the weak auxin response mutants *ibr5-1* and *tir1-1* (Figure 10D). Intriguingly, *ein2-1* failed to suppress ABA-resistant seed germination of the strong auxin transport mutant *aux1-7* or the pleiotropic mutant *axr1-3* (Figure 10D). The additive nature of combining altered ethylene production or response with altered auxin response on ABA-responsive seed germination suggests that auxin and ethylene independently affect ABA regulation of seed germination.

## Discussion

### Whole genome sequencing to identify causative mutations

In this study, we demonstrate the ease of using whole genome sequencing of backcrossed bulk segregants to quickly identify causative mutations. We found that sequencing backcrossed bulk segregants, rather than outcrossed bulk segregants, facilitates identification of a small interval of candidate mutations (Table 2 and Thole and Strader, unpublished data). We were curious how many mutations would be present without backcrossing and therefore sequenced nonbackcrossed pooled M_4_ seedlings for AR241 and AR211. For each of these mutants, homozygous EMS-caused mutations were littered throughout the genome, with 144 mutations identified in AR241 and 195 mutations identified in AR211. We then used recombination mapping to determine which of these mutations were linked to the ABA resistance phenotype. Although we were successful in identifying the causative mutations by sequencing nonbackcrossed mutant lines, we have decided to use sequencing of backcrossed bulk segregants in the future for mutant identification because the small number of homozygous mutations in these pools facilitates identification of the causative mutation.

### Disruption of many genes can result in reduced ABA responsiveness in root elongation assays

Although ABA has long been known to inhibit root elongation, our understanding of mechanisms regulating ABA inhibition of root elongation remains incomplete. As may be expected, many components of the ABA signaling pathway are required for ABA inhibition of root elongation. For example, the *pyr1 pyl1 pyl2 pyl4* quadruple mutant of ABA receptors displays strong ABA resistance in root elongation (Park *et al.* 2009) and the gain-of-function *abi1-1* and *abi2-1* mutations in protein phosphatases required for ABA perception display strong ABA resistant root elongation (Koornneef *et al.* 1984) and higher order loss-of-function mutations in this gene family results in ABA hypersensitivity in root elongation inhibition (Rubio *et al.* 2009). Downstream of ABA perception, the SnRK2.2 and SnRK2.3 protein kinases (Fujii *et al.* 2007), the ABA INSENSITIVE5 transcription factor (Lopez-Molina *et al.* 2001), and the calcium-dependent protein kinases CPK4 and CPK11 (Zhu *et al.* 2007) regulate ABA-responsive inhibition of root elongation. Additionally, G-proteins (Pandey *et al.* 2006) and the nuclear protein X1 (Kim *et al.* 2009) negatively regulate the inhibitory effects of ABA on root elongation and PROLINE-RICH EXTENSIN-LIKE RECEPTOR KINASE4 (Bai *et al.* 2009), and the NADPH oxidases AtrbohD and AtrbohF (Jiao *et al.* 2013) positively regulate ABA-responsive inhibition of root elongation.

To identify additional factors required for ABA-responsive inhibition of root elongation, we screened for mutations that conferred ABA-resistant root elongation (AR mutants). In this study, we characterized the ABA responsiveness of 21 AR lines and found that we could separate ABA-responsive root elongation inhibition from ABA-responsive seed germination inhibition in our mutants. In our screen, we identified alleles of *ABI2* (Figure 2), *EIN2* (Figure 2), *AUX1* (Figure 2 and Figure 3), *AXR4* (Figure 4), *EIR1*/*PIN2* (Figure 5), and *IAA16* (Rinaldi *et al.* 2012) that could confer ABA resistance in root elongation assays.

EIN2 is a positive regulator of ethylene responses. The N-terminus of ER-associated EIN2 (Bisson *et al.* 2009) contains 12 transmembrane helices and shows weak homology to mammalian NRAMP (natural resistance-associated macrophage protein) metal transporters, whereas its cytosolic C-terminus carries a nuclear localization signal. CTR1-dependent phosphorylation of EIN2 (Chen *et al.* 2011; Ju *et al.* 2012) determines whether the C-terminus of EIN2 is cleaved from its N-terminal transmembrane domain to allow translocation to the nucleus (Ju *et al.* 2012; Qiao *et al.* 2012; Wen *et al.* 2012) for activation of downstream ethylene-response transcription factors. *EIN2* alleles have previously been identified in screens for enhanced response to ABA (*era3*) (Ghassemian *et al.* 2000) and for suppression of *abi1-1* (Beaudoin *et al.* 2000). Interestingly, *ein2* mutants display both hypersensitivity to ABA in seed germination assays and resistance to ABA in root elongation assays (Figure 7 and Figure 8) (Beaudoin *et al.* 2000; Ghassemian *et al.* 2000), suggesting that ethylene dampens ABA-responsive inhibition of seed germination and enhances ABA-responsive inhibition of root elongation. Thus far, molecular connections between ethylene effects on ABA responsiveness are not well-characterized.

We identified two alleles of *auxin resistant1* (*aux1*) in our screen for mutants displaying resistance to the inhibitory effects of ABA on root elongation (Figure 2 and Figure 3). AUX1 is a major influx carrier of auxin; therefore, *aux1* mutants are defective in many processes that require auxin transport, including ethylene responses (Stepanova and Alonso 2009; Muday *et al.* 2012) and ABA responses. *aux1* displays resistance to the ABA-inducible ProDc3:GUS expression (Rock and Sun 2005), to the inhibitory effects of ABA on root elongation (Strader *et al.* 2008a), and to the inhibitory effects of ABA on postgerminative growth (Belin *et al.* 2009), suggesting that auxin transport is necessary for ABA-responsive inhibition of these processes.

We also identified an allele of *auxin resistant4* (*aux4*) in our screen (Figure 4). The ER-localized AXR4 protein is necessary for targeting the AUX1 auxin influx carrier to the plasma membrane (Dharmasiri *et al.* 2006). *axr4* displays resistance to ABA-inducible ProDc3:GUS expression (Rock and Sun 2005) and mild resistance to the inhibitory effects of ABA on root elongation (Figure 1A and Figure 4C) (Hobbie and Estelle 1994). Additionally, *axr4* displays an interaction with ABA by principle component analysis of root system architecture (Ristova *et al.* 2013). Because AXR4 is required for proper AUX1 targeting to the plasma membrane, we would expect *axr4* mutants to phenocopy *aux1* mutants. Intriguingly, unlike the *aux1* mutant or many other auxin resistant mutants, *axr4* displays wild-type sensitivity to the ethylene precursor ACC in root elongation assays (Figure 6A) (Hobbie and Estelle 1994), suggesting that AXR4 is not required for AUX1 activity in response to ethylene.

EIR1/PIN2 is a polarly localized auxin efflux carrier (Feraru and Friml 2008). We identified an allele of *ethylene insensitive root1*/*pin-formed2* (*eir1/pin2*) in our screen for mutants displaying resistance to the inhibitory effects of ABA on root elongation (Figure 4). *eir1* displays strong resistance to the inhibitory effects of ABA on postgerminative growth (Belin *et al.* 2009) and moderate resistance to the inhibitory effects of ABA on root elongation (Figure 1A and Figure 5C) and seed germination (Figure 1B), consistent with the possibility that auxin efflux is necessary for ABA-responsive inhibition of these processes. In addition, *eir1*/*pin2* was previously described for resistance to the growth-inhibitory effects of ethylene (Roman *et al.* 1995; Luschnig *et al.* 1998), suggesting that EIR1 is required for ethylene responses, in addition to being required for ABA responses. Indeed, auxin transport from the root meristem to the root elongation zone is required for ethylene responses (Stepanova and Alonso 2009; Muday *et al.* 2012) and also appears to be required for the inhibitory effects of ABA on root elongation.

Because we identified components of the ABA, ethylene, and auxin pathways, the remaining unidentified AR mutants could represent factors in the ABA, auxin, or ethylene signaling cascades or in an unanticipated response pathway. In particular, it will be interesting to further characterize class 2 AR mutants (Table 1), which appear to play stronger roles in responses to the inhibitory effects of ABA on root elongation than on seed germination.

### Auxin–ethylene epistasis in ABA response

Because we identified factors required for both auxin and ethylene response in our screen for factors necessary for ABA-responsive root elongation, we became interested in understanding the auxin–ethylene interaction in modulating ABA responses. Previous auxin–ethylene interaction studies have been complicated by auxin-related ethylene inhibitor off-target effects; the ethylene biosynthesis inhibitor aminoethyoxyvinylglycine (AVG) also inhibits auxin biosynthesis (Soeno *et al.* 2010), whereas the ethylene receptor inhibitor AgNO_3_ promotes auxin efflux independently of its effects on ethylene responsiveness (Strader *et al.* 2009). To circumvent these issues, we examined double mutants between the ethylene overproducing mutant *eto1* with auxin-resistant mutants (Strader *et al.* 2010) and double mutants between the strong ethylene-resistant mutant *ein2* and auxin-resistant mutants. We used these double mutants to examine auxin–ethylene epistasis in modulating ABA-responsive root elongation and ABA-responsive seed germination.

Decreased auxin or ethylene sensitivity results in decreased ABA-responsive inhibition of root elongation. Because double mutants between *ein2* and auxin-resistant mutants do not display additive phenotypes (Figure 9), auxin and ethylene likely act in a linear pathway to affect ABA-responsive inhibition of primary root elongation. In addition, ethylene overproduction by *eto1* did not compensate for the reduced ABA sensitivity displayed by auxin-resistant mutants (Figure 9), suggesting that auxin acts downstream of ethylene in regulating ABA-responsive root elongation inhibition. This suggestion that auxin and ethylene act in a linear pathway to affect ABA-responsive root elongation is consistent with other reports that auxin acts downstream of ethylene to regulate root cell expansion (Strader *et al.* 2010) and that root ethylene responses require intact auxin responses (Růžička *et al.* 2007; Stepanova *et al.* 2007).

Decreased auxin responsiveness results in decreased ABA responsiveness in seed germination inhibition assays (Figure 8) (Monroe-Augustus *et al.* 2003; Belin *et al.* 2009; Rinaldi *et al.* 2012), whereas decreased ethylene responsiveness results in increased ABA responsiveness in seed germination inhibition assays (Figure 8) (Beaudoin *et al.* 2000; Ghassemian *et al.* 2000; Subbiah and Reddy 2010). Conversely, increasing ethylene production results in decreased ABA responsiveness in seed germination inhibition assays (Beaudoin *et al.* 2000; Ghassemian *et al.* 2000; Subbiah and Reddy 2010). The additive nature of combining altered ethylene production or response with altered auxin response or transport on ABA-responsive seed germination (Figure 10) suggests that auxin and ethylene independently affect ABA regulation of seed germination. Future experiments to uncover the molecular nature of these complex interactions should provide interesting insight into differential hormone crosstalk at various developmental time points and tissue types.

## Supplementary Material

Supporting Information
