# Supplementary material for: Abscisic Acid Regulates Root Elongation Through the Activities of Auxin and Ethylene in Arabidopsis thaliana
Source: G3 (Bethesda). 2014 May 15;4(7):1259–74. doi: 10.1534/g3.114.011080 (PMC4455775; doi:10.1534/g3.114.011080)
Supplement: Supporting Information [file supp_4_7_1259__index.html]

Abscisic Acid Regulates Root Elongation Through the Activities of Auxin and Ethylene in Arabidopsis thaliana — Supporting Information 

# Abscisic Acid Regulates Root Elongation Through the Activities of Auxin and Ethylene in *Arabidopsis thaliana*

## Supporting Information for Thole *et al.*, 2014

**Files in this Data Supplement:**

- Supporting Information - Tables S1-S3 (PDF, 188 KB)
- Table S1 - Homozygous EMS-related mutations in the AR241 Exome. (PDF, 122 KB)
- Table S2 - Genotyping markers used to positionally clone AR241 and AR211. (PDF, 124 KB)
- Table S3 - Homozygous EMS-related mutations in the AR211 Exome. (PDF, 124 KB)
