# Supplementary material for: Abscisic Acid Regulates Root Elongation Through the Activities of Auxin and Ethylene in Arabidopsis thaliana
Source: G3 (Bethesda). 2014 May 15;4(7):1259–74. doi: 10.1534/g3.114.011080 (PMC4455775; doi:10.1534/g3.114.011080)
Supplement: Supporting Information [file supp_g3.114.011080_011080SI.pdf]

G3 · Genes | Genomes | Genetics

Abscisic acid regulates root elongation through the activities of auxin and ethylene in *Arabidopsis thaliana*

Julie M. Thole[1], Erin R. Beisner[2], James Liu[3], Savina V. Venkova[4], and Lucia C. Strader[5]

Department of Biology, Washington University in St. Louis, St. Louis, Missouri 63130

[1]*Present address: Department of Biology, Saint Louis University, St.Louis, Missouri 63103*
[2]*Present address: Department of Medicinal Chemistry and Pharmacognosy, University of Illinois at Chicago, Chicago, IL 60607*
[3]*Present address: The University of Texas Health Science Center, San Antonio, TX 78229*
[4]*Department of Biochemistry and Cell Biology, Rice University, Houston, Texas 77005*

[5]*Corresponding author:* Department of Biology, CB-1137, Washington University, One Brookings Drive, St. Louis, MO 63130.
Email: strader@wustl.edu

DOI: 10.1534/g3.114.011080

Table S1 Homozygous EMS-related mutations in the AR241 Exome

Chromosome	Location[1]	Reference sequence	Mutant sequence	Gene	Amino acid change	Codon change
1	570351	G	A	AT1G02650	C/Y	tGt/tAt
1	776720	G	A	AT1G03190	G/E	gGa/gAa
1	792780	G	A	AT1G03240	A/V	gCg/gTg
1	1317235	G	A	AT1G04700	R/Q	cGg/cAg
1	1470175	G	A	AT1G05100	G/E	gGa/gAa
1	1751949	G	A	AT1G05820	V/I	Gtt/Att
1	2385274	G	A	AT1G07705	E/K	Gaa/Aaa
1	2812322	G	A	AT1G08790	D/N	Gac/Aac
1	2927915	G	A	AT1G09070	G/D	gGc/gAc
1	3033900	G	A	AT1G09400	P/S	Cct/Tct
1	3266046	G	A	AT1G10010	G/D	gGt/gAt
1	3367220	G	A	AT1G10280	R/C	Cgc/Tgc
1	3691108	G	A	AT1G11070	P/L	cCg/cTg
1	3818161	G	A	AT1G11350	S/L	tCa/tTa
1	4014976	G	A	AT1G11905	A/T	Gct/Act
1	4173407	G	A	AT1G12270	G/S	Ggt/Agt
1	4956980	G	A	AT1G14480	A/T	Gca/Aca
1	5427361	G	A	AT1G15770	P/S	Cct/Tct
1	5639610	G	A	AT1G16500	S/N	aGc/aAc
1	6099926	G	A	AT1G17730	E/K	Gaa/Aaa
1	6590873	G	A	AT1G19090	G/E	gGg/gAg
1	6969768	G	A	AT1G20100	P/L	cCg/cTg
1	7488007	G	A	AT1G21380	A/V	gCt/gTt
1	8065783	G	A	AT1G22770	C/Y	tGt/tAt
1	8241286	G	A	AT1G23210	E/K	Gag/Aag
1	10104331	G	A	AT1G28760	G/R	Gga/Aga
1	10217836	G	A	AT1G29240	P/S	Cca/Tca
1	12088324	G	A	AT1G33340	D/N	Gat/Aat
1	12163493	G	A	AT1G33540	R/*	Cga/Tga
1	12463233	G	A	AT1G34220	Q/*	Caa/Taa
1	13026656	G	A	AT1G35420	D/N	Gat/Aat
1	16654369	G	A	AT1G43900	G/S	Ggt/Agt
1	18018428	G	A	AT1G48720	G/D	gGt/gAt
1	18936189	G	A	AT1G51110	G/E	gGa/gAa
1	19059085	G	A	AT1G51405	E/K	Gaa/Aaa
1	20064561	G	A	AT1G53730	R/H	cGc/cAc
1	20512340	G	A	AT1G54990	W/*	tGg/tAg
1	20661588	G	A	AT1G55350	P/L	cCa/cTa
1	21405591	G	A	AT1G57790	L/F	Ctt/Ttt
1	21875436	G	A	AT1G59540	G/E	gGa/gAa

1	22066012	G	A	AT1G59940	S/L	tCg/tTg
1	22428699	G	A	AT1G60913	P/L	cCc/cTc
1	23882511	G	A	AT1G64350	E/K	Gag/Aag
1	25147642	G	A	AT1G67220	A/T	Gcg/Acg
1	25539907	G	A	AT1G68140	P/S	Cct/Tct
1	25868125	G	A	AT1G68820	E/K	Gaa/Aaa
1	26361358	G	A	AT1G69990	A/V	gCt/gTt
1	26558750	G	A	AT1G70460	G/E	gGg/gAg
1	27345276	G	A	AT1G72630	E/K	Gaa/Aaa
1	27413235	G	A	AT1G72840	T/M	aCg/aTg
1	27916739	G	A	AT1G74230	G/D	gGc/gAc
1	28208461	G	A	AT1G75150	P/S	Cct/Tct
1	29682303	G	A	AT1G78940	T/I	aCc/aTc
1	30090745	G	A	AT1G79990	A/T	Gca/Aca
2	2426951	G	A	AT2G06200	G/E	gGa/gAa
2	3623765	G	A	AT2G08986	V/M	Gtg/Atg
2	7518468	C	T	AT2G17290	A/V	gCt/gTt
2	7607536	C	T	AT2G17500	L/F	Ctt/Ttt
2	7748009	C	T	AT2G17820	R/Q	cGa/cAa
2	7819170	C	T	AT2G17970	W/*	tgG/tgA
2	7892943	C	T	AT2G18150	R/K	aGa/aAa
2	7999983	C	T	AT2G18450	D/N	Gat/Aat
2	8009749	C	T	AT2G18480	A/T	Gcc/Acc
2	8076473	C	T	AT2G18610	G/R	Ggg/Agg
2	8408865	C	T	AT2G19410	D/N	Gat/Aat
2	8508595	C	T	AT2G19710	P/L	cCt/cTt
2	8632519	C	T	AT2G20000	G/D	gGt/gAt
2	8846325	C	T	AT2G20550	R/Q	cGa/cAa
2	9117277	C	T	AT2G21300	E/K	Gaa/Aaa
2	10137070	C	T	AT2G23810	V/I	Gtt/Att
2	10418528	C	T	AT2G24520	P/S	Cca/Tca
2	11202418	C	T	AT2G26310	P/S	Cct/Tct
2	11670172	C	T	AT2G27260	A/V	gCt/gTt
2	11804031	C	T	AT2G27680	D/N	Gat/Aat
2	13016895	C	T	AT2G30550	R/W	Cgg/Tgg
2	13272020	C	T	AT2G31141	E/K	Gaa/Aaa
2	13474785	C	T	AT2G31680	G/D	gGt/gAt
2	13945640	C	T	AT2G32870	W/*	tgG/tgA
2	15025390	C	T	AT2G35738	L/F	Ctt/Ttt
2	16045457	C	T	AT2G38300	G/R	Gga/Aga
3	124806	G	A	AT3G01330	L/F	Ctt/Ttt
3	725973	G	A	AT3G03140	R/Q	cGg/cAg
3	1088180	G	A	AT3G04140	A/T	Gct/Act

3	2182741	G	A	AT3G06920	R/K	aGa/aAa
3	2238539	G	A	AT3G07070	V/M	Gtg/Atg
3	2687551	G	A	AT3G08850	R/W	Cgg/Tgg
3	2757810	G	A	AT3G09032	T/I	aCt/aTt
3	2874014	G	A	AT3G09360	E/K	Gag/Aag
3	4164574	G	A	AT3G13010	P/S	Cca/Tca
3	5044688	G	A	AT3G14980	L/F	Ctc/Ttc
3	5287390	G	A	AT3G15604	T/M	aCg/aTg
3	7913271	G	A	AT3G22380	D/N	Gat/Aat
3	8055053	G	A	AT3G22790	R/C	Cgt/Tgt
3	8346080	G	A	AT3G23325	R/K	aGa/aAa
3	9007127	G	A	AT3G24670	S/L	tCg/tTg
3	9264939	G	A	AT3G25510	P/S	Cca/Tca
3	10424860	G	A	AT3G28030	D/N	Gat/Aat
3	11597068	G	A	AT3G29765	P/S	Cca/Tca
3	17921885	G	A	AT3G48390	A/T	Gct/Act
3	17936902	G	A	AT3G48430	V/M	Gtg/Atg
3	18923361	G	A	AT3G50920	S/F	tCt/tTt
3	19070082	G	A	AT3G51370	S/N	aGt/aAt
3	19159614	G	A	AT3G51650	V/I	Gtt/Att
3	20448362	G	A	AT3G55160	L/F	Ctt/Ttt
3	21426904	G	A	AT3G57860	G/S	Ggc/Agc
3	21517030	G	A	AT3G58110	D/N	Gac/Aac
3	22149325	G	A	AT3G59960	G/S	Ggt/Agt
3	22445157	G	A	AT3G60730	G/E	gGg/gAg
3	23030506	G	A	AT3G62220	R/C	Cgt/Tgt
3	23433011	G	A	AT3G63460	L/F	Ctc/Ttc
4	2675681	G	A	AT4G05190	D/N	Gat/Aat
4	2807348	G	A	AT4G05520	G/R	Gga/Aga
4	6870005	G	A	AT4G11290	G/R	Gga/Aga
4	6998720	G	A	AT4G11550	T/I	aCc/aTc
4	7987196	G	A	AT4G13750	E/K	Gaa/Aaa
4	8025494	G	A	AT4G13870	L/F	Ctc/Ttc
4	8172233	G	A	AT4G14160	W/*	tgG/tgA
4	8172233	G	A	AT4G14160	A/T	Gct/Act
4	8759449	G	A	AT4G15340	P/L	cCt/cTt
4	9177893	G	A	AT4G16210	S/F	tCt/tTt
4	10454957	G	A	AT4G19110	A/V	gCt/gTt
4	11347534	G	A	AT4G21326	V/M	Gtg/Atg
4	11392826	G	A	AT4G21380	P/S	Cct/Tct
4	11455610	G	A	AT4G21530	E/K	Gag/Aag
5	569994	G	A	AT5G02540	D/N	Gat/Aat
5	1622198	G	A	AT5G05470	S/N	aGc/aAc

5	1716162	G	A	AT5G05710	V/I	Gtc/Atc
5	2680964	G	A	AT5G08330	R/H	cGt/cAt
5	2787213	G	A	AT5G08600	E/K	Gag/Aag
5	3328122	G	A	AT5G10540	A/T	Gct/Act
5	3636793	G	A	AT5G11400	P/S	Cca/Tca
5	4847383	G	A	AT5G14970	A/T	Gca/Aca
5	4872733	G	A	AT5G15050	L/F	Ctc/Ttc
5	5311884	G	A	AT5G16260	W/*	tgG/tgA
5	8600423	G	A	AT5G24960	A/V	gCa/gTa
5	8649053	G	A	AT5G25100	A/V	gCc/gTc
5	9460685	G	A	AT5G26890	W/*	tGg/tAg
5	10880071	G	A	AT5G28850	P/S	Cct/Tct
5	12264824	G	A	AT5G32613	C/Y	tGc/tAc
5	14029924	G	A	AT5G35910	R/W	Cgg/Tgg
5	14854345	G	A	AT5G37450	P/S	Cct/Tct
5	15285471	G	A	AT5G38260	P/S	Cca/Tca
5	20049111	G	A	AT5G49440	R/K	aGa/aAa
5	20425018	G	A	AT5G50170	G/E	gGa/gAa

[1]Basepair location on chromosome 2 in TAIR v10

Table S2 Genotyping markers used to positionally clone AR241 and AR211.

Positional markers for AR241 mutations

Gene	Enzyme	Wt	AR241	Oligonucleotides[a]
At1g54990	M*fe*I	235, 30	265	ATGATTCAGCTTTAGGGTTAGCTTC**C**AATT<br>AGCAACAACAGCTTCTCTCC
At2g17500	E*co*RI	239, 29	268	CATGGGACTTACATTTCTAATCGGAGGA**A**TT<br>TCCTCATCGCAGATTGCTG
At3g08850	H*inf*I	152, 153	305	TGATGTTCGGTCACCTGAAC<br>CGAAATTCTTCAAACCCGAACC
At4g19110	B*sm*I	331, 28	359	GTCACTCGTAGTAGTGTATCCGAGA**A**TG<br>ATTGACGAGGCAAAGACGC
At5G08330	N*de*I	267	130, 137	ACAACGACGGAGCAGTGAG<br>AGTAGTGCCAGTTCCAGTGG
At5g28850	H*inf*I	242	148, 94	GCATCGGGTTCTAATTCTGCC<br>AGAAATCAGTGAGTTAACTCCATTCC

Positional markers for AR211 mutations

Gene	Enzyme	Wt	AR211	Oligonucleotides[a]
At1g10940	B*sp*HI	211	178, 33	AGTTGCCAGGCTCATGAAG<br>GTTAAACACCCAATCAGCTAACG
At1g29540	H*inf*I	130, 151	281	CCTAAGGCACAGAGCAACAC<br>GGAAGAAGAGGAAGAATGAAGCC
At1g58250	B*gl*II	211, 30	241	TTAAAGATGTTGAAGCATCTCGAATGAGAT<br>GATGTTCTTCTCAACTCCTGAGC
At1g70950	M*se*I	229	200, 29	AGAGTCAATGTGAGTTTGATGAGGAAA**T**T<br>TGTTCATCGAGTTTCGATCCC
At2g24850	F*ok*I	159, 174	333	GGTCGCGGAGATGGCTAG<br>ATTGCTTGAACTACCTGTTGGTG
At2g44760	M*se*I	290	225, 65	GAAATGGCGGAGAAGGATAAGC<br>TCCTACACCTTTCCAAATCCC
At3g01300	D*de*I	262	138, 124	CTACAACTAGCAATGCAGAAAGC<br>TGACCCTGAAGGCCATCAG
At3g03405	H*inf*I	130, 166	296	GGAGAATCTATCTACACAAACTGTTGG<br>CATATTGCATGAGATGGGAGAAACC
At3g16880	A*ci*I	282	166, 116	CAGTAACACTAGAGATGTGGTTTGG<br>AGAATTTCCTTTCACAGAGACATCC
At3g21430	F*ok*I	200, 30	230	TAGTGCCGTGGAAAAGCAGCAAAAAGGAT<br>GAGACTAGAACACACCTGCAG
At3g49060	αT*aq*I	241, 30	211	CACTGAGAAGACTGGTTAAAGGAGAGA**T**CG<br>CCATATACATAAGATCCTTTACCCGAC
At3g56600	H*ae*III	177.96	273	TCTAACGGAGTTGGAGCTGC<br>AAGACTAAGCCCTGCAGCAG
At4g01670	A*cc*I	352, 30	382	TGAGCTTGAACCGGGTCGGGTTTCGTAT**A**<br>GCCCATATTTTAATTCCCCACC
At4g09870	H*inf*I	234	204, 30	GGAAATCGTATGCCTCTTCCC<br>CGACAAAAAAACTTAGTCCAGGGCAA**G**AA
At4g12120	M*fe*I	150, 171	321	TGCAGATGTATGTCGTGCC<br>GCTCACTGTACTGAGGCAAAG
At4g13370	A*lu*I	250	165, 85	CGCTTGGAATTCGCTACTCC<br>GAGCAAGCGCTTGTCTCAC
At4g24800	X*ho*I	221, 30	251	GACATTTTGAATGAATATGTGGAGACT**C**GA<br>CAAGAGCAAGATCATCAAGGC
At5g15730	H*inf*I	321	191, 130	TGGAACAACCACACCAAAGATC

				GGGAAATGAGTGGATCTGTACAC
At5g26990	H*ae*III	187, 30	217	GAGAGAGTTTCCTGATGGAAATTTTCAG**G**<br>TGGAAGCATACCTTTCAGGC
At5g40360	B*sp*HI	300	270, 30	ATAAATATTCGTAGGTTGTTGGTGAGAAT**C**<br>GTTGCCAACTATCTTGTGGACTTC
At5g46040	H*py*188I	230, 30	260	ACCCATAAATGGAGGCTCTGTACTATC**T**CA<br>GCATTGAGAATGTTGTGAAGCC
At5g53120	H*py*188I	82, 185	267	GACGACAGTACTCATTCTCTTCC<br>TTCTCGTTCTAGACGACGAGG
At5g56450	H*py*188I	300	270, 30	ATGATGAAGTGAAGAGGTTCTTGAATT**C**GG<br>TTGAGCTTAGACCAATCCATGTC
At5g57090	A*ci*I	217, 230	247	TCCTCAAGGAATCGTTCCTTTTGTTT**C**CG<br>TTACAGGCAAAGCAACCAGC
At5g66140	B*gl*II	230	200, 30	TACCCAAAGTGGTGGTGTCAGACCCTT**A**G<br>CAGAGCACGGATAGCGAG

[a]Underlined nucleotide is the introduced mutation for this dCAPS genotyping marker (MICHAELS AND AMASINO 1998; NEFF *et al.* 1998).

Table S3 Homozygous EMS-related mutations in the AR211 Exome

Chromosome	Location[1]	Reference sequence	Mutant sequence	Gene	Amino acid change	Codon change
1	139060	C	T	AT1G01355	P/S	Cct/Tct
1	561276	C	T	AT1G02630	A/T	Gct/Act
1	723055	C	T	AT1G03060	A/T	Gcg/Acg
1	734475	C	T	AT1G03080	D/N	Gat/Aat
1	960025	C	T	AT1G03820	E/K	Gaa/Aaa
1	1186179	C	T	AT1G04400	G/D	gGt/gAt
1	1411381	C	T	AT1G04970	L/F	Ctc/Ttc
1	1913814	C	T	AT1G06250	G/E	gGa/gAa
1	3658079	G	A	AT1G10940	A/V	gCc/gTc
1	4732699	G	A	AT1G13800	R/C	Cgc/Tgc
1	5390306	G	A	AT1G15670	R/Q	cGg/cAg
1	5677353	G	A	AT1G16610	L/F	Ctt/Ttt
1	5809513	G	A	AT1G16980	S/N	aGt/aAt
1	6313539	G	A	AT1G18340	L/F	Ctt/Ttt
1	6362408	G	A	AT1G18480	G/E	gGg/gAg
1	7101970	G	A	AT1G20500	P/L	cCa/cTa
1	7650632	G	A	AT1G21760	R/H	cGc/cAc
1	8223327	G	A	AT1G23190	R/H	cGt/cAt
1	9118563	G	A	AT1G26355	L/F	Ctt/Ttt
1	9183098	G	A	AT1G26570	A/T	Gct/Act
1	10030338	G	A	AT1G28520	E/K	Gaa/Aaa
1	10327509	G	A	AT1G29540	R/K	aGa/aAa
1	11146522	G	A	AT1G31190	A/T	Gct/Act
1	11858257	G	A	AT1G32760	G/S	Ggt/Agt
1	12379443	G	A	AT1G34042	S/F	tCc/tTc
1	12745064	G	A	AT1G34760	L/F	Ctt/Ttt
1	16277445	G	A	AT1G43190	R/C	Cgt/Tgt
1	17320715	G	A	AT1G47250	D/N	Gat/Aat
1	18105816	G	A	AT1G48940	L/F	Ctt/Ttt
1	18220800	G	A	AT1G49250	G/D	gGc/gAc
1	18679322	G	A	AT1G50420	L/F	Ctt/Ttt
1	19113399	G	A	AT1G51538	E/K	Gaa/Aaa
1	19778471	G	A	AT1G53070	G/D	gGc/gAc
1	19795263	G	A	AT1G53130	S/F	tCc/tTc
1	20080338	G	A	AT1G53790	P/S	Ccg/Tcg
1	20222720	G	A	AT1G54170	S/L	tCa/tTa
1	20865721	G	A	AT1G55820	D/N	Gat/Aat
1	21029118	G	A	AT1G56190	G/S	Ggt/Agt
1	21588781	G	A	AT1G58250	S/F	tCt/tTt
1	21594500	G	A	AT1G58250	P/S	Cca/Tca
1	21762177	G	A	AT1G58602	R/K	aGa/aAa
1	22144577	G	A	AT1G60070	S/F	tCt/tTt
1	23117273	G	A	AT1G62450	P/L	cCg/cTg
1	23936314	G	A	AT1G64430	R/K	aGa/aAa
1	24287328	G	A	AT1G65380	S/N	aGt/aAt
1	24461387	G	A	AT1G65770	H/Y	Cat/Tat
1	24930277	G	A	AT1G66820	G/E	gGa/gAa
1	24943705	G	A	AT1G66860	A/T	Gct/Act
1	25711879	G	A	AT1G68526	P/L	cCg/cTg
1	25904516	G	A	AT1G68890	A/T	Gca/Aca

1	25923434	G	A	AT1G68940	L/F	Ctt/Ttt
1	26753628	G	A	AT1G70950	D/N	Gat/Aat
1	26755012	G	A	AT1G70950	E/K	Gag/Aag
1	26815470	G	A	AT1G71100	A/T	Gcg/Acg
1	27198112	G	A	AT1G72250	W/*	tgG/tgA
1	28031987	G	A	AT1G74630	W/*	tgG/tgA
1	28114701	G	A	AT1G74830	L/F	Ctt/Ttt
1	29456040	G	A	AT1G78280	A/T	Gct/Act
1	29972916	G	A	AT1G79650	L/F	Ctt/Ttt
2	9529495	C	T	AT2G22440	L/F	Ctc/Ttc
2	10584074	C	T	AT2G24850	G/R	Gga/Aga
2	11627352	C	T	AT2G27200	P/L	cCc/cTc
2	11815196	C	T	AT2G27700	M/I	atG/atA
2	13004796	C	T	AT2G30520	D/N	Gac/Aac
2	13347629	C	T	AT2G31300	G/S	Ggt/Agt
2	13604529	C	T	AT2G31970	S/F	tCc/tTc
2	14229078	C	T	AT2G33610	P/L	cCc/cTc
2	14267486	C	T	AT2G33730	R/K	aGa/aAa
2	14428394	C	T	AT2G34170	L/F	Ctc/Ttc
2	15174068	C	T	AT2G36180	G/D	gGt/gAt
2	15335766	C	T	AT2G36570	P/S	Cca/Tca
2	15405954	C	T	AT2G36730	G/D	gGt/gAt
2	17729309	C	T	AT2G42580	S/F	tCt/tTt
2	18047784	C	T	AT2G43465	G/E	gGa/gAa
2	18454443	C	T	AT2G44760	G/S	Ggt/Agt
2	18665617	C	T	AT2G45260	A/T	Gct/Act
2	18738519	C	T	AT2G45460	R/Q	cGa/cAa
2	19401957	C	T	AT2G47250	G/R	Gga/Aga
2	19478628	C	T	AT2G47460	R/C	Cgt/Tgt
3	92622	C	T	AT3G01300	G/R	Gga/Aga
3	331987	C	T	AT3G01990	S/F	tCt/tTt
3	625634	C	T	AT3G02860	V/I	Gtt/Att
3	792554	C	T	AT3G03350	L/F	Ctt/Ttt
3	809888	C	T	AT3G03405	R/Q	cGa/cAa
3	1497902	C	T	AT3G05260	A/T	Gcg/Acg
3	1532686	C	T	AT3G05360	S/N	aGc/aAc
3	1721097	C	T	AT3G05790	A/T	Gcg/Acg
3	2783357	C	T	AT3G09090	E/K	Gag/Aag
3	3835399	C	T	AT3G12040	A/T	Gca/Aca
3	4252189	C	T	AT3G13222	E/K	Gaa/Aaa
3	5097716	C	T	AT3G15130	E/K	Gaa/Aaa
3	5462454	C	T	AT3G16110	E/K	Gag/Aag
3	5723741	C	T	AT3G16810	A/T	Gcg/Acg
3	5767183	C	T	AT3G16880	R/C	Cgc/Tgc
3	6112950	C	T	AT3G17850	D/N	Gat/Aat
3	6193177	C	T	AT3G18080	L/F	Ctt/Ttt
3	6276323	C	T	AT3G18290	S/F	tCt/tTt
3	6384180	C	T	AT3G18550	P/S	Cca/Tca
3	7550168	C	T	AT3G21430	R/K	aGa/aAa
3	8179479	C	T	AT3G23020	G/R	Ggg/Agg
3	8617009	C	T	AT3G23860	V/I	Gtt/Att
3	8806375	C	T	AT3G24300	G/E	gGg/gAg
3	9661903	C	T	AT3G26380	P/S	Cct/Tct
3	9792146	C	T	AT3G26630	G/E	gGa/gAa

3	9915941	C	T	AT3G26910	A/T	Gca/Aca
3	10985368	C	T	AT3G28960	G/S	Ggt/Agt
3	11729342	C	T	AT3G29810	T/I	aCt/aTt
3	18188669	C	T	AT3G49060	G/E	gGa/gAa
3	18528294	C	T	AT3G49970	A/T	Gca/Aca
3	18694933	C	T	AT3G50380	D/N	Gat/Aat
3	19895848	C	T	AT3G53680	A/T	Gca/Aca
3	20706123	C	T	AT3G55780	S/F	tCt/tTt
3	20969987	C	T	AT3G56600	P/S	Cct/Tct
4	37193	G	A	AT4G00100	G/R	Gga/Aga
4	203800	G	A	AT4G00450	G/E	gGg/gAg
4	269723	G	A	AT4G00650	G/E	gGg/gAg
4	419631	G	A	AT4G00970	D/N	Gat/Aat
4	711931	G	A	AT4G01670	S/F	tCc/tTc
4	894797	G	A	AT4G02030	E/K	Gag/Aag
4	6200956	G	A	AT4G09870	G/D	gGt/gAt
4	6299728	G	A	AT4G10070	T/M	aCg/aTg
4	6416798	G	A	AT4G10350	P/L	cCt/cTt
4	6783448	G	A	AT4G11130	D/N	Gac/Aac
4	6988567	G	A	AT4G11530	R/K	aGg/aAg
4	7258522	G	A	AT4G12120	Q/*	Caa/Taa
4	7315373	G	A	AT4G12320	L/F	Ctt/Ttt
4	7409891	G	A	AT4G12490	S/F	tCt/tTt
4	7614091	G	A	AT4G13040	E/K	Gaa/Aaa
4	7779985	G	A	AT4G13370	P/L	cCg/cTg
4	9052848	G	A	AT4G15975	G/E	gGa/gAa
4	9067901	G	A	AT4G16008	E/K	Gaa/Aaa
4	9810849	G	A	AT4G17610	P/S	Cca/Tca
4	9923398	G	A	AT4G17850	D/N	Gat/Aat
4	9942510	G	A	AT4G17895	E/K	Gaa/Aaa
4	10373483	G	A	AT4G18940	D/N	Gac/Aac
4	10746919	G	A	AT4G19750	T/I	aCc/aTc
4	10988610	G	A	AT4G20350	R/K	aGa/aAa
4	11157007	G	A	AT4G20830	G/R	Gga/Aga
4	11577582	G	A	AT4G21820	A/T	Gct/Act
4	12700257	G	A	AT4G24600	V/M	Gtg/Atg
4	12783682	G	A	AT4G24800	E/K	Gag/Aag
4	13302839	G	A	AT4G26270	S/F	tCt/tTt
4	13922668	G	A	AT4G27980	S/N	aGc/aAc
4	15434283	G	A	AT4G31900	A/T	Gca/Aca
4	15445308	G	A	AT4G31920	S/F	tCt/tTt
4	16918583	G	A	AT4G35680	A/T	Gca/Aca
4	17942085	G	A	AT4G38280	D/N	Gat/Aat
5	429342	G	A	AT5G02170	G/R	Gga/Aga
5	1068539	G	A	AT5G03960	P/S	Ccg/Tcg
5	1209773	G	A	AT5G04320	E/K	Gag/Aag
5	1372241	G	A	AT5G04750	A/T	Gct/Act
5	1941097	G	A	AT5G06350	R/H	cGt/cAt
5	2127579	G	A	AT5G06850	S/F	tCt/tTt
5	2344427	G	A	AT5G07400	R/K	aGa/aAa
5	2350767	G	A	AT5G07420	A/T	Gcg/Acg
5	2411992	G	A	AT5G07630	R/Q	cGg/cAg
5	2744997	G	A	AT5G08480	G/D	gGc/gAc
5	2883284	G	A	AT5G09290	E/K	Gag/Aag

5	3026507	G	A	AT5G09750	P/L	cCg/cTg
5	3551582	G	A	AT5G11160	G/D	gGc/gAc
5	3784369	G	A	AT5G11740	V/I	Gtc/Atc
5	3805942	G	A	AT5G11800	A/V	gCt/gTt
5	3863428	G	A	AT5G11970	T/M	aCg/aTg
5	3937794	G	A	AT5G12180	G/E	gGg/gAg
5	4756942	G	A	AT5G14730	D/N	Gat/Aat
5	4959462	G	A	AT5G15270	G/E	gGa/gAa
5	5131730	G	A	AT5G15730	G/E	gGa/gAa
5	5253530	G	A	AT5G16080	S/F	tCc/tTc
5	5872278	G	A	AT5G17790	P/S	Cca/Tca
5	6136517	G	A	AT5G18490	S/F	tCc/tTc
5	6447214	G	A	AT5G19170	G/D	gGc/gAc
5	6687316	G	A	AT5G19780	M/I	atG/atA
5	6716199	G	A	AT5G19870	L/F	Ctt/Ttt
5	7376608	G	A	AT5G22290	A/V	gCc/gTc
5	7918346	G	A	AT5G23480	D/N	Gat/Aat
5	8052616	G	A	AT5G23880	V/I	Gtc/Atc
5	9493106	G	A	AT5G26990	S/N	aGc/aAc
5	9571457	G	A	AT5G27200	A/T	Gct/Act
5	9822363	G	A	AT5G27730	G/D	gGt/gAt
5	10780921	G	A	AT5G28740	E/K	Gag/Aag
5	14720407	G	A	AT5G37180	G/S	Ggt/Agt
5	15281640	G	A	AT5G38250	A/V	gCt/gTt
5	16145916	G	A	AT5G40360	V/M	Gtg/Atg
5	16853788	G	A	AT5G42180	D/N	Gat/Aat
5	18672194	C	T	AT5G46040	E/K	Gaa/Aaa
5	18931148	C	T	AT5G46650	R/W	Cgg/Tgg
5	21182641	C	T	AT5G52130	E/K	Gag/Aag
5	22858782	C	T	AT5G56450	G/E	gGa/gAa
5	23097145	C	T	AT5G57070	P/S	Cct/Tct
5	23104210	C	T	AT5G57090	A/V	gCc/gTc
5	24115961	C	T	AT5G59870	A/T	Gcc/Acc
5	25166804	C	T	AT5G62680	G/E	gGg/gAg
5	25685612	C	T	AT5G64210	R/K	aGa/aAa
5	26052558	C	T	AT5G65207	E/K	Gag/Aag
5	26437907	C	T	AT5G66140	G/D	gGt/gAt

[1]Basepair location on chromosome 2 in TAIR v10
