# Supplementary material for: Abscisic Acid Regulates Root Elongation Through the Activities of Auxin and Ethylene in Arabidopsis thaliana
Source: G3 (Bethesda). 2014 May 15;4(7):1259–74. doi: 10.1534/g3.114.011080 (PMC4455775; doi:10.1534/g3.114.011080)
Supplement: Supporting Information [file supp_g3.114.011080_TableS1.pdf]

Table S1 Homozygous EMS-related mutations in the AR241 Exome

Chromosome	Location[1]	Reference sequence	Mutant sequence	Gene	Amino acid change	Codon change
1	570351	G	A	AT1G02650	C/Y	tGt/tAt
1	776720	G	A	AT1G03190	G/E	gGa/gAa
1	792780	G	A	AT1G03240	A/V	gCg/gTg
1	1317235	G	A	AT1G04700	R/Q	cGg/cAg
1	1470175	G	A	AT1G05100	G/E	gGa/gAa
1	1751949	G	A	AT1G05820	V/I	Gtt/Att
1	2385274	G	A	AT1G07705	E/K	Gaa/Aaa
1	2812322	G	A	AT1G08790	D/N	Gac/Aac
1	2927915	G	A	AT1G09070	G/D	gGc/gAc
1	3033900	G	A	AT1G09400	P/S	Cct/Tct
1	3266046	G	A	AT1G10010	G/D	gGt/gAt
1	3367220	G	A	AT1G10280	R/C	Cgc/Tgc
1	3691108	G	A	AT1G11070	P/L	cCg/cTg
1	3818161	G	A	AT1G11350	S/L	tCa/tTa
1	4014976	G	A	AT1G11905	A/T	Gct/Act
1	4173407	G	A	AT1G12270	G/S	Ggt/Agt
1	4956980	G	A	AT1G14480	A/T	Gca/Aca
1	5427361	G	A	AT1G15770	P/S	Cct/Tct
1	5639610	G	A	AT1G16500	S/N	aGc/aAc
1	6099926	G	A	AT1G17730	E/K	Gaa/Aaa
1	6590873	G	A	AT1G19090	G/E	gGg/gAg
1	6969768	G	A	AT1G20100	P/L	cCg/cTg
1	7488007	G	A	AT1G21380	A/V	gCt/gTt
1	8065783	G	A	AT1G22770	C/Y	tGt/tAt
1	8241286	G	A	AT1G23210	E/K	Gag/Aag
1	10104331	G	A	AT1G28760	G/R	Gga/Aga
1	10217836	G	A	AT1G29240	P/S	Cca/Tca
1	12088324	G	A	AT1G33340	D/N	Gat/Aat
1	12163493	G	A	AT1G33540	R/*	Cga/Tga
1	12463233	G	A	AT1G34220	Q/*	Caa/Taa
1	13026656	G	A	AT1G35420	D/N	Gat/Aat
1	16654369	G	A	AT1G43900	G/S	Ggt/Agt
1	18018428	G	A	AT1G48720	G/D	gGt/gAt
1	18936189	G	A	AT1G51110	G/E	gGa/gAa
1	19059085	G	A	AT1G51405	E/K	Gaa/Aaa
1	20064561	G	A	AT1G53730	R/H	cGc/cAc
1	20512340	G	A	AT1G54990	W/*	tGg/tAg
1	20661588	G	A	AT1G55350	P/L	cCa/cTa
1	21405591	G	A	AT1G57790	L/F	Ctt/Ttt
1	21875436	G	A	AT1G59540	G/E	gGa/gAa

1	22066012	G	A	AT1G59940	S/L	tCg/tTg
1	22428699	G	A	AT1G60913	P/L	cCc/cTc
1	23882511	G	A	AT1G64350	E/K	Gag/Aag
1	25147642	G	A	AT1G67220	A/T	Gcg/Acg
1	25539907	G	A	AT1G68140	P/S	Cct/Tct
1	25868125	G	A	AT1G68820	E/K	Gaa/Aaa
1	26361358	G	A	AT1G69990	A/V	gCt/gTt
1	26558750	G	A	AT1G70460	G/E	gGg/gAg
1	27345276	G	A	AT1G72630	E/K	Gaa/Aaa
1	27413235	G	A	AT1G72840	T/M	aCg/aTg
1	27916739	G	A	AT1G74230	G/D	gGc/gAc
1	28208461	G	A	AT1G75150	P/S	Cct/Tct
1	29682303	G	A	AT1G78940	T/I	aCc/aTc
1	30090745	G	A	AT1G79990	A/T	Gca/Aca
2	2426951	G	A	AT2G06200	G/E	gGa/gAa
2	3623765	G	A	AT2G08986	V/M	Gtg/Atg
2	7518468	C	T	AT2G17290	A/V	gCt/gTt
2	7607536	C	T	AT2G17500	L/F	Ctt/Ttt
2	7748009	C	T	AT2G17820	R/Q	cGa/cAa
2	7819170	C	T	AT2G17970	W/*	tgG/tgA
2	7892943	C	T	AT2G18150	R/K	aGa/aAa
2	7999983	C	T	AT2G18450	D/N	Gat/Aat
2	8009749	C	T	AT2G18480	A/T	Gcc/Acc
2	8076473	C	T	AT2G18610	G/R	Ggg/Agg
2	8408865	C	T	AT2G19410	D/N	Gat/Aat
2	8508595	C	T	AT2G19710	P/L	cCt/cTt
2	8632519	C	T	AT2G20000	G/D	gGt/gAt
2	8846325	C	T	AT2G20550	R/Q	cGa/cAa
2	9117277	C	T	AT2G21300	E/K	Gaa/Aaa
2	10137070	C	T	AT2G23810	V/I	Gtt/Att
2	10418528	C	T	AT2G24520	P/S	Cca/Tca
2	11202418	C	T	AT2G26310	P/S	Cct/Tct
2	11670172	C	T	AT2G27260	A/V	gCt/gTt
2	11804031	C	T	AT2G27680	D/N	Gat/Aat
2	13016895	C	T	AT2G30550	R/W	Cgg/Tgg
2	13272020	C	T	AT2G31141	E/K	Gaa/Aaa
2	13474785	C	T	AT2G31680	G/D	gGt/gAt
2	13945640	C	T	AT2G32870	W/*	tgG/tgA
2	15025390	C	T	AT2G35738	L/F	Ctt/Ttt
2	16045457	C	T	AT2G38300	G/R	Gga/Aga
3	124806	G	A	AT3G01330	L/F	Ctt/Ttt
3	725973	G	A	AT3G03140	R/Q	cGg/cAg
3	1088180	G	A	AT3G04140	A/T	Gct/Act

3	2182741	G	A	AT3G06920	R/K	aGa/aAa
3	2238539	G	A	AT3G07070	V/M	Gtg/Atg
3	2687551	G	A	AT3G08850	R/W	Cgg/Tgg
3	2757810	G	A	AT3G09032	T/I	aCt/aTt
3	2874014	G	A	AT3G09360	E/K	Gag/Aag
3	4164574	G	A	AT3G13010	P/S	Cca/Tca
3	5044688	G	A	AT3G14980	L/F	Ctc/Ttc
3	5287390	G	A	AT3G15604	T/M	aCg/aTg
3	7913271	G	A	AT3G22380	D/N	Gat/Aat
3	8055053	G	A	AT3G22790	R/C	Cgt/Tgt
3	8346080	G	A	AT3G23325	R/K	aGa/aAa
3	9007127	G	A	AT3G24670	S/L	tCg/tTg
3	9264939	G	A	AT3G25510	P/S	Cca/Tca
3	10424860	G	A	AT3G28030	D/N	Gat/Aat
3	11597068	G	A	AT3G29765	P/S	Cca/Tca
3	17921885	G	A	AT3G48390	A/T	Gct/Act
3	17936902	G	A	AT3G48430	V/M	Gtg/Atg
3	18923361	G	A	AT3G50920	S/F	tCt/tTt
3	19070082	G	A	AT3G51370	S/N	aGt/aAt
3	19159614	G	A	AT3G51650	V/I	Gtt/Att
3	20448362	G	A	AT3G55160	L/F	Ctt/Ttt
3	21426904	G	A	AT3G57860	G/S	Ggc/Agc
3	21517030	G	A	AT3G58110	D/N	Gac/Aac
3	22149325	G	A	AT3G59960	G/S	Ggt/Agt
3	22445157	G	A	AT3G60730	G/E	gGg/gAg
3	23030506	G	A	AT3G62220	R/C	Cgt/Tgt
3	23433011	G	A	AT3G63460	L/F	Ctc/Ttc
4	2675681	G	A	AT4G05190	D/N	Gat/Aat
4	2807348	G	A	AT4G05520	G/R	Gga/Aga
4	6870005	G	A	AT4G11290	G/R	Gga/Aga
4	6998720	G	A	AT4G11550	T/I	aCc/aTc
4	7987196	G	A	AT4G13750	E/K	Gaa/Aaa
4	8025494	G	A	AT4G13870	L/F	Ctc/Ttc
4	8172233	G	A	AT4G14160	W/*	tgG/tgA
4	8172233	G	A	AT4G14160	A/T	Gct/Act
4	8759449	G	A	AT4G15340	P/L	cCt/cTt
4	9177893	G	A	AT4G16210	S/F	tCt/tTt
4	10454957	G	A	AT4G19110	A/V	gCt/gTt
4	11347534	G	A	AT4G21326	V/M	Gtg/Atg
4	11392826	G	A	AT4G21380	P/S	Cct/Tct
4	11455610	G	A	AT4G21530	E/K	Gag/Aag
5	569994	G	A	AT5G02540	D/N	Gat/Aat
5	1622198	G	A	AT5G05470	S/N	aGc/aAc

5	1716162	G	A	AT5G05710	V/I	Gtc/Atc
5	2680964	G	A	AT5G08330	R/H	cGt/cAt
5	2787213	G	A	AT5G08600	E/K	Gag/Aag
5	3328122	G	A	AT5G10540	A/T	Gct/Act
5	3636793	G	A	AT5G11400	P/S	Cca/Tca
5	4847383	G	A	AT5G14970	A/T	Gca/Aca
5	4872733	G	A	AT5G15050	L/F	Ctc/Ttc
5	5311884	G	A	AT5G16260	W/*	tgG/tgA
5	8600423	G	A	AT5G24960	A/V	gCa/gTa
5	8649053	G	A	AT5G25100	A/V	gCc/gTc
5	9460685	G	A	AT5G26890	W/*	tGg/tAg
5	10880071	G	A	AT5G28850	P/S	Cct/Tct
5	12264824	G	A	AT5G32613	C/Y	tGc/tAc
5	14029924	G	A	AT5G35910	R/W	Cgg/Tgg
5	14854345	G	A	AT5G37450	P/S	Cct/Tct
5	15285471	G	A	AT5G38260	P/S	Cca/Tca
5	20049111	G	A	AT5G49440	R/K	aGa/aAa
5	20425018	G	A	AT5G50170	G/E	gGa/gAa

[1]Basepair location on chromosome 2 in TAIR v10
