# Supplementary material for: Abscisic Acid Regulates Root Elongation Through the Activities of Auxin and Ethylene in Arabidopsis thaliana
Source: G3 (Bethesda). 2014 May 15;4(7):1259–74. doi: 10.1534/g3.114.011080 (PMC4455775; doi:10.1534/g3.114.011080)
Supplement: Supporting Information [file supp_g3.114.011080_TableS2.pdf]

Table S2 Genotyping markers used to positionally clone AR241 and AR211.

Positional markers for AR241 mutations

Gene	Enzyme	Wt	AR241	Oligonucleotides[a]
At1g54990	M*fe*I	235, 30	265	ATGATTCAGCTTTAGGGTTAGCTTC**C**AATT<br>AGCAACAACAGCTTCTCTCC
At2g17500	E*co*RI	239, 29	268	CATGGGACTTACATTTCTAATCGGAGGA**A**TT<br>TCCTCATCGCAGATTGCTG
At3g08850	H*inf*I	152, 153	305	TGATGTTCGGTCACCTGAAC<br>CGAAATTCTTCAAACCCGAACC
At4g19110	B*sm*I	331, 28	359	GTCACTCGTAGTAGTGTATCCGAGA**A**TG<br>ATTGACGAGGCAAAGACGC
At5G08330	N*de*I	267	130, 137	ACAACGACGGAGCAGTGAG<br>AGTAGTGCCAGTTCCAGTGG
At5g28850	H*inf*I	242	148, 94	GCATCGGGTTCTAATTCTGCC<br>AGAAATCAGTGAGTTAACTCCATTCC

Positional markers for AR211 mutations

Gene	Enzyme	Wt	AR211	Oligonucleotides[a]
At1g10940	B*sp*HI	211	178, 33	AGTTGCCAGGCTCATGAAG<br>GTTAAACACCCAATCAGCTAACG
At1g29540	H*inf*I	130, 151	281	CCTAAGGCACAGAGCAACAC<br>GGAAGAAGAGGAAGAATGAAGCC
At1g58250	B*gl*II	211, 30	241	TTAAAGATGTTGAAGCATCTCGAATGAGAT<br>GATGTTCTTCTCAACTCCTGAGC
At1g70950	M*se*I	229	200, 29	AGAGTCAATGTGAGTTTGATGAGGAAA**T**T<br>TGTTCATCGAGTTTCGATCCC
At2g24850	F*ok*I	159, 174	333	GGTCGCGGAGATGGCTAG<br>ATTGCTTGAACTACCTGTTGGTG
At2g44760	M*se*I	290	225, 65	GAAATGGCGGAGAAGGATAAGC<br>TCCTACACCTTTCCAAATCCC
At3g01300	D*de*I	262	138, 124	CTACAACTAGCAATGCAGAAAGC<br>TGACCCTGAAGGCCATCAG
At3g03405	H*inf*I	130, 166	296	GGAGAATCTATCTACACAAACTGTTGG<br>CATATTGCATGAGATGGGAGAAACC
At3g16880	A*ci*I	282	166, 116	CAGTAACACTAGAGATGTGGTTTGG<br>AGAATTTCCTTTCACAGAGACATCC
At3g21430	F*ok*I	200, 30	230	TAGTGCCGTGGAAAAGCAGCAAAAAGGAT<br>GAGACTAGAACACACCTGCAG
At3g49060	α*Taq*I	241, 30	211	CACTGAGAAGACTGGTTAAAGGAGAGA**T**CG<br>CCATATACATAAGATCCTTTACCCGAC
At3g56600	H*ae*III	177.96	273	TCTAACGGAGTTGGAGCTGC<br>AAGACTAAGCCCTGCAGCAG
At4g01670	A*cc*I	352, 30	382	TGAGCTTGAACCGGGTCGGGTTTCGTAT**A**<br>GCCCATATTTTAATTCCCCACC
At4g09870	H*inf*I	234	204, 30	GGAAATCGTATGCCTCTTCCC<br>CGACAAAAAAACTTAGTCCAGGGCAA**G**AA
At4g12120	M*fe*I	150, 171	321	TGCAGATGTATGTCGTGCC<br>GCTCACTGTACTGAGGCAAAG
At4g13370	A*lu*I	250	165, 85	CGCTTGGAATTCGCTACTCC<br>GAGCAAGCGCTTGTCTCAC
At4g24800	X*ho*I	221, 30	251	GACATTTTGAATGAATATGTGGAGACT**C**GA<br>CAAGAGCAAGATCATCAAGGC
At5g15730	H*inf*I	321	191, 130	TGGAACAACCACACCAAAGATC

				GGGAAATGAGTGGATCTGTACAC
At5g26990	H*ae*III	187, 30	217	GAGAGAGTTTCCTGATGGAAATTTTCAG**G**
				TGGAAGCATACCTTTCAGGC
At5g40360	B*sp*HI	300	270, 30	ATAAATATTCGTAGGTTGTTGGTGAGAAT**C**
				GTTGCCAACTATCTTGTGGACTTC
At5g46040	H*py*188I	230, 30	260	ACCCATAAATGGAGGCTCTGTACTATC**T**CA
				GCATTGAGAATGTTGTGAAGCC
At5g53120	H*py*188I	82, 185	267	GACGACAGTACTCATTCTCTTCC
				TTCTCGTTCTAGACGACGAGG
At5g56450	H*py*188I	300	270, 30	ATGATGAAGTGAAGAGGTTCTTGAATT**C**GG
				TTGAGCTTAGACCAATCCATGTC
At5g57090	A*ci*I	217, 230	247	TCCTCAAGGAATCGTTCCTTTTGTTT**C**CG
				TTACAGGCAAAGCAACCAGC
At5g66140	B*gl*II	230	200, 30	TACCCAAAGTGGTGGTGTCAGACCCTT**A**G
				CAGAGCACGGATAGCGAG

[a]Underlined nucleotide is the introduced mutation for this dCAPS genotyping marker (MICHAELS AND AMASINO 1998; NEFF *et al.* 1998).
