# Supplementary material for: Abscisic Acid Regulates Root Elongation Through the Activities of Auxin and Ethylene in Arabidopsis thaliana
Source: G3 (Bethesda). 2014 May 15;4(7):1259–74. doi: 10.1534/g3.114.011080 (PMC4455775; doi:10.1534/g3.114.011080)
Supplement: Supporting Information [file supp_g3.114.011080_TableS3.pdf]

Table S3 Homozygous EMS-related mutations in the AR211 Exome

Chromosome	Location[1]	Reference sequence	Mutant sequence	Gene	Amino acid change	Codon change
1	139060	C	T	AT1G01355	P/S	Cct/Tct
1	561276	C	T	AT1G02630	A/T	Gct/Act
1	723055	C	T	AT1G03060	A/T	Gcg/Acg
1	734475	C	T	AT1G03080	D/N	Gat/Aat
1	960025	C	T	AT1G03820	E/K	Gaa/Aaa
1	1186179	C	T	AT1G04400	G/D	gGt/gAt
1	1411381	C	T	AT1G04970	L/F	Ctc/Ttc
1	1913814	C	T	AT1G06250	G/E	gGa/gAa
1	3658079	G	A	AT1G10940	A/V	gCc/gTc
1	4732699	G	A	AT1G13800	R/C	Cgc/Tgc
1	5390306	G	A	AT1G15670	R/Q	cGg/cAg
1	5677353	G	A	AT1G16610	L/F	Ctt/Ttt
1	5809513	G	A	AT1G16980	S/N	aGt/aAt
1	6313539	G	A	AT1G18340	L/F	Ctt/Ttt
1	6362408	G	A	AT1G18480	G/E	gGg/gAg
1	7101970	G	A	AT1G20500	P/L	cCa/cTa
1	7650632	G	A	AT1G21760	R/H	cGc/cAc
1	8223327	G	A	AT1G23190	R/H	cGt/cAt
1	9118563	G	A	AT1G26355	L/F	Ctt/Ttt
1	9183098	G	A	AT1G26570	A/T	Gct/Act
1	10030338	G	A	AT1G28520	E/K	Gaa/Aaa
1	10327509	G	A	AT1G29540	R/K	aGa/aAa
1	11146522	G	A	AT1G31190	A/T	Gct/Act
1	11858257	G	A	AT1G32760	G/S	Ggt/Agt
1	12379443	G	A	AT1G34042	S/F	tCc/tTc
1	12745064	G	A	AT1G34760	L/F	Ctt/Ttt
1	16277445	G	A	AT1G43190	R/C	Cgt/Tgt
1	17320715	G	A	AT1G47250	D/N	Gat/Aat
1	18105816	G	A	AT1G48940	L/F	Ctt/Ttt
1	18220800	G	A	AT1G49250	G/D	gGc/gAc
1	18679322	G	A	AT1G50420	L/F	Ctt/Ttt
1	19113399	G	A	AT1G51538	E/K	Gaa/Aaa
1	19778471	G	A	AT1G53070	G/D	gGc/gAc
1	19795263	G	A	AT1G53130	S/F	tCc/tTc
1	20080338	G	A	AT1G53790	P/S	Ccg/Tcg
1	20222720	G	A	AT1G54170	S/L	tCa/tTa
1	20865721	G	A	AT1G55820	D/N	Gat/Aat
1	21029118	G	A	AT1G56190	G/S	Ggt/Agt
1	21588781	G	A	AT1G58250	S/F	tCt/tTt
1	21594500	G	A	AT1G58250	P/S	Cca/Tca
1	21762177	G	A	AT1G58602	R/K	aGa/aAa
1	22144577	G	A	AT1G60070	S/F	tCt/tTt
1	23117273	G	A	AT1G62450	P/L	cCg/cTg
1	23936314	G	A	AT1G64430	R/K	aGa/aAa
1	24287328	G	A	AT1G65380	S/N	aGt/aAt
1	24461387	G	A	AT1G65770	H/Y	Cat/Tat
1	24930277	G	A	AT1G66820	G/E	gGa/gAa
1	24943705	G	A	AT1G66860	A/T	Gct/Act
1	25711879	G	A	AT1G68526	P/L	cCg/cTg
1	25904516	G	A	AT1G68890	A/T	Gca/Aca

1	25923434	G	A	AT1G68940	L/F	Ctt/Ttt
1	26753628	G	A	AT1G70950	D/N	Gat/Aat
1	26755012	G	A	AT1G70950	E/K	Gag/Aag
1	26815470	G	A	AT1G71100	A/T	Gcg/Acg
1	27198112	G	A	AT1G72250	W/*	tgG/tgA
1	28031987	G	A	AT1G74630	W/*	tgG/tgA
1	28114701	G	A	AT1G74830	L/F	Ctt/Ttt
1	29456040	G	A	AT1G78280	A/T	Gct/Act
1	29972916	G	A	AT1G79650	L/F	Ctt/Ttt
2	9529495	C	T	AT2G22440	L/F	Ctc/Ttc
2	10584074	C	T	AT2G24850	G/R	Gga/Aga
2	11627352	C	T	AT2G27200	P/L	cCc/cTc
2	11815196	C	T	AT2G27700	M/I	atG/atA
2	13004796	C	T	AT2G30520	D/N	Gac/Aac
2	13347629	C	T	AT2G31300	G/S	Ggt/Agt
2	13604529	C	T	AT2G31970	S/F	tCc/tTc
2	14229078	C	T	AT2G33610	P/L	cCc/cTc
2	14267486	C	T	AT2G33730	R/K	aGa/aAa
2	14428394	C	T	AT2G34170	L/F	Ctc/Ttc
2	15174068	C	T	AT2G36180	G/D	gGt/gAt
2	15335766	C	T	AT2G36570	P/S	Cca/Tca
2	15405954	C	T	AT2G36730	G/D	gGt/gAt
2	17729309	C	T	AT2G42580	S/F	tCt/tTt
2	18047784	C	T	AT2G43465	G/E	gGa/gAa
2	18454443	C	T	AT2G44760	G/S	Ggt/Agt
2	18665617	C	T	AT2G45260	A/T	Gct/Act
2	18738519	C	T	AT2G45460	R/Q	cGa/cAa
2	19401957	C	T	AT2G47250	G/R	Gga/Aga
2	19478628	C	T	AT2G47460	R/C	Cgt/Tgt
3	92622	C	T	AT3G01300	G/R	Gga/Aga
3	331987	C	T	AT3G01990	S/F	tCt/tTt
3	625634	C	T	AT3G02860	V/I	Gtt/Att
3	792554	C	T	AT3G03350	L/F	Ctt/Ttt
3	809888	C	T	AT3G03405	R/Q	cGa/cAa
3	1497902	C	T	AT3G05260	A/T	Gcg/Acg
3	1532686	C	T	AT3G05360	S/N	aGc/aAc
3	1721097	C	T	AT3G05790	A/T	Gcg/Acg
3	2783357	C	T	AT3G09090	E/K	Gag/Aag
3	3835399	C	T	AT3G12040	A/T	Gca/Aca
3	4252189	C	T	AT3G13222	E/K	Gaa/Aaa
3	5097716	C	T	AT3G15130	E/K	Gaa/Aaa
3	5462454	C	T	AT3G16110	E/K	Gag/Aag
3	5723741	C	T	AT3G16810	A/T	Gcg/Acg
3	5767183	C	T	AT3G16880	R/C	Cgc/Tgc
3	6112950	C	T	AT3G17850	D/N	Gat/Aat
3	6193177	C	T	AT3G18080	L/F	Ctt/Ttt
3	6276323	C	T	AT3G18290	S/F	tCt/tTt
3	6384180	C	T	AT3G18550	P/S	Cca/Tca
3	7550168	C	T	AT3G21430	R/K	aGa/aAa
3	8179479	C	T	AT3G23020	G/R	Ggg/Agg
3	8617009	C	T	AT3G23860	V/I	Gtt/Att
3	8806375	C	T	AT3G24300	G/E	gGg/gAg
3	9661903	C	T	AT3G26380	P/S	Cct/Tct
3	9792146	C	T	AT3G26630	G/E	gGa/gAa

3	9915941	C	T	AT3G26910	A/T	Gca/Aca
3	10985368	C	T	AT3G28960	G/S	Ggt/Agt
3	11729342	C	T	AT3G29810	T/I	aCt/aTt
3	18188669	C	T	AT3G49060	G/E	gGa/gAa
3	18528294	C	T	AT3G49970	A/T	Gca/Aca
3	18694933	C	T	AT3G50380	D/N	Gat/Aat
3	19895848	C	T	AT3G53680	A/T	Gca/Aca
3	20706123	C	T	AT3G55780	S/F	tCt/tTt
3	20969987	C	T	AT3G56600	P/S	Cct/Tct
4	37193	G	A	AT4G00100	G/R	Gga/Aga
4	203800	G	A	AT4G00450	G/E	gGg/gAg
4	269723	G	A	AT4G00650	G/E	gGg/gAg
4	419631	G	A	AT4G00970	D/N	Gat/Aat
4	711931	G	A	AT4G01670	S/F	tCc/tTc
4	894797	G	A	AT4G02030	E/K	Gag/Aag
4	6200956	G	A	AT4G09870	G/D	gGt/gAt
4	6299728	G	A	AT4G10070	T/M	aCg/aTg
4	6416798	G	A	AT4G10350	P/L	cCt/cTt
4	6783448	G	A	AT4G11130	D/N	Gac/Aac
4	6988567	G	A	AT4G11530	R/K	aGg/aAg
4	7258522	G	A	AT4G12120	Q/*	Caa/Taa
4	7315373	G	A	AT4G12320	L/F	Ctt/Ttt
4	7409891	G	A	AT4G12490	S/F	tCt/tTt
4	7614091	G	A	AT4G13040	E/K	Gaa/Aaa
4	7779985	G	A	AT4G13370	P/L	cCg/cTg
4	9052848	G	A	AT4G15975	G/E	gGa/gAa
4	9067901	G	A	AT4G16008	E/K	Gaa/Aaa
4	9810849	G	A	AT4G17610	P/S	Cca/Tca
4	9923398	G	A	AT4G17850	D/N	Gat/Aat
4	9942510	G	A	AT4G17895	E/K	Gaa/Aaa
4	10373483	G	A	AT4G18940	D/N	Gac/Aac
4	10746919	G	A	AT4G19750	T/I	aCc/aTc
4	10988610	G	A	AT4G20350	R/K	aGa/aAa
4	11157007	G	A	AT4G20830	G/R	Gga/Aga
4	11577582	G	A	AT4G21820	A/T	Gct/Act
4	12700257	G	A	AT4G24600	V/M	Gtg/Atg
4	12783682	G	A	AT4G24800	E/K	Gag/Aag
4	13302839	G	A	AT4G26270	S/F	tCt/tTt
4	13922668	G	A	AT4G27980	S/N	aGc/aAc
4	15434283	G	A	AT4G31900	A/T	Gca/Aca
4	15445308	G	A	AT4G31920	S/F	tCt/tTt
4	16918583	G	A	AT4G35680	A/T	Gca/Aca
4	17942085	G	A	AT4G38280	D/N	Gat/Aat
5	429342	G	A	AT5G02170	G/R	Gga/Aga
5	1068539	G	A	AT5G03960	P/S	Ccg/Tcg
5	1209773	G	A	AT5G04320	E/K	Gag/Aag
5	1372241	G	A	AT5G04750	A/T	Gct/Act
5	1941097	G	A	AT5G06350	R/H	cGt/cAt
5	2127579	G	A	AT5G06850	S/F	tCt/tTt
5	2344427	G	A	AT5G07400	R/K	aGa/aAa
5	2350767	G	A	AT5G07420	A/T	Gcg/Acg
5	2411992	G	A	AT5G07630	R/Q	cGg/cAg
5	2744997	G	A	AT5G08480	G/D	gGc/gAc
5	2883284	G	A	AT5G09290	E/K	Gag/Aag

5	3026507	G	A	AT5G09750	P/L	cCg/cTg
5	3551582	G	A	AT5G11160	G/D	gGc/gAc
5	3784369	G	A	AT5G11740	V/I	Gtc/Atc
5	3805942	G	A	AT5G11800	A/V	gCt/gTt
5	3863428	G	A	AT5G11970	T/M	aCg/aTg
5	3937794	G	A	AT5G12180	G/E	gGg/gAg
5	4756942	G	A	AT5G14730	D/N	Gat/Aat
5	4959462	G	A	AT5G15270	G/E	gGa/gAa
5	5131730	G	A	AT5G15730	G/E	gGa/gAa
5	5253530	G	A	AT5G16080	S/F	tCc/tTc
5	5872278	G	A	AT5G17790	P/S	Cca/Tca
5	6136517	G	A	AT5G18490	S/F	tCc/tTc
5	6447214	G	A	AT5G19170	G/D	gGc/gAc
5	6687316	G	A	AT5G19780	M/I	atG/atA
5	6716199	G	A	AT5G19870	L/F	Ctt/Ttt
5	7376608	G	A	AT5G22290	A/V	gCc/gTc
5	7918346	G	A	AT5G23480	D/N	Gat/Aat
5	8052616	G	A	AT5G23880	V/I	Gtc/Atc
5	9493106	G	A	AT5G26990	S/N	aGc/aAc
5	9571457	G	A	AT5G27200	A/T	Gct/Act
5	9822363	G	A	AT5G27730	G/D	gGt/gAt
5	10780921	G	A	AT5G28740	E/K	Gag/Aag
5	14720407	G	A	AT5G37180	G/S	Ggt/Agt
5	15281640	G	A	AT5G38250	A/V	gCt/gTt
5	16145916	G	A	AT5G40360	V/M	Gtg/Atg
5	16853788	G	A	AT5G42180	D/N	Gat/Aat
5	18672194	C	T	AT5G46040	E/K	Gaa/Aaa
5	18931148	C	T	AT5G46650	R/W	Cgg/Tgg
5	21182641	C	T	AT5G52130	E/K	Gag/Aag
5	22858782	C	T	AT5G56450	G/E	gGa/gAa
5	23097145	C	T	AT5G57070	P/S	Cct/Tct
5	23104210	C	T	AT5G57090	A/V	gCc/gTc
5	24115961	C	T	AT5G59870	A/T	Gcc/Acc
5	25166804	C	T	AT5G62680	G/E	gGg/gAg
5	25685612	C	T	AT5G64210	R/K	aGa/aAa
5	26052558	C	T	AT5G65207	E/K	Gag/Aag
5	26437907	C	T	AT5G66140	G/D	gGt/gAt

[1]Basepair location on chromosome 2 in TAIR v10
